# Machine learning for patient risk stratification: standing on, or looking over, the shoulders of clinicians?

**DOI:** 10.1038/s41746-021-00426-3

**Published:** 2021-03-30

**Authors:** Brett K. Beaulieu-Jones, William Yuan, Gabriel A. Brat, Andrew L. Beam, Griffin Weber, Marshall Ruffin, Isaac S. Kohane

**Affiliations:** 1 Salutary Inc., Tysons, VA USA; 2 Department of Biomedical Informatics, Harvard Medical School, Boston, MA USA; 3 Department of Epidemiology, Harvard T.H. Chan School of Public Health, Boston, MA USA

**Keywords:** Prognosis, Predictive markers

## Abstract

Machine learning can help clinicians to make individualized patient predictions only if researchers demonstrate models that contribute novel insights, rather than learning the most likely next step in a set of actions a clinician will take. We trained deep learning models using only clinician-initiated, administrative data for 42.9 million admissions using three subsets of data: demographic data only, demographic data and information available at admission, and the previous data plus charges recorded during the first day of admission. Models trained on charges during the first day of admission achieve performance close to published full EMR-based benchmarks for inpatient outcomes: inhospital mortality (0.89 AUC), prolonged length of stay (0.82 AUC), and 30-day readmission rate (0.71 AUC). Similar performance between models trained with only clinician-initiated data and those trained with full EMR data purporting to include information about patient state and physiology should raise concern in the deployment of these models. Furthermore, these models exhibited significant declines in performance when evaluated over only myocardial infarction (MI) patients relative to models trained over MI patients alone, highlighting the importance of physician diagnosis in the prognostic performance of these models. These results provide a benchmark for predictive accuracy trained only on prior clinical actions and indicate that models with similar performance may derive their signal by looking over clinician’s shoulders—using clinical behavior as the expression of preexisting intuition and suspicion to generate a prediction. For models to guide clinicians in individual decisions, performance exceeding these benchmarks is necessary.

## Introduction

Machine learning for healthcare promises to have a major impact on the delivery of data-driven personalized medicine^1,2^. One of the applications with the widest potential is patient risk stratification (i.e., diagnosis, prognosis)^3^. Individualized patient risk stratification requires machine learning models to predict the future disease state of a patient based on his or her current clinical state and available history^4^. However, understanding whether predictions are based on physician behavior rather than faithful representations of patient physiology is critical for identifying which applications of these predictions will be sound.

**Fig. 1:**
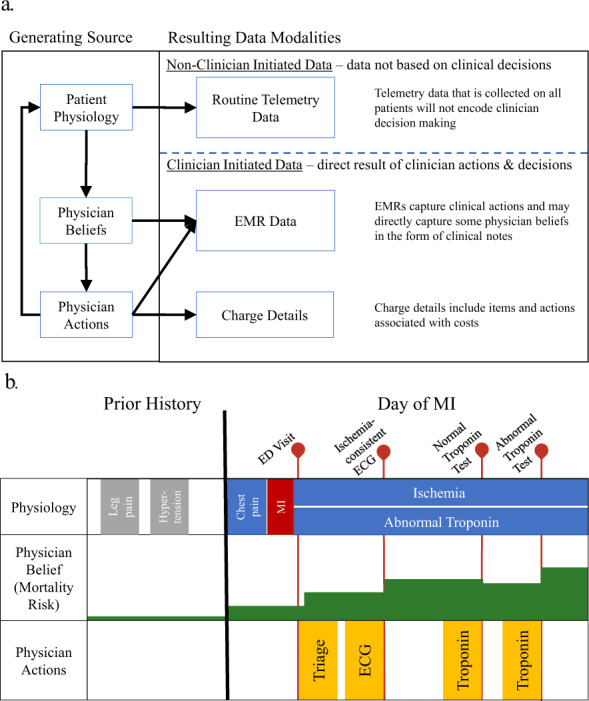
Clinician-initiated data alone is a filtered representation of patient physiology. **a** Clinician-initiated and non-clinician-initiated data are distinguished by their proximity as readouts of patient physiology, as well as the presence of the expertise of the clinician. **b** Physician actions are a reflection of their beliefs regarding a patient, which are formed through examination of patient physiology.

When a patient’s physiology reaches a state requiring examination, the clinician’s beliefs regarding potential patient outcomes are updated, which then inform which actions the clinician chooses to make (or not make). These actions, in turn, influence the patient’s resulting physiology, and the cycle repeats (Fig. 1a). Consequently, we define data present in the EMR as one of two categories: “clinician-initiated” data and “non-clinician-initiated data”. Clinician-initiated data are data elements created through specific actions (or inactions) or insights of the clinician. Clinician-initiated data include expressions of physician decision making, including test orders, prescriptions, diagnoses, referrals, consultations, or procedure orders that are not routine for the patient population in question. Non-clinician-initiated data include routine orders and direct physiological measurements of the patient because insights from the clinician are not involved in the creation of these data. This is a nuanced distinction because the presence of a lab test can be but is not always clinician-initiated, while the outputs of that lab test are always non-clinician-initiated data. The conscious order of a nonroutine lab test represents a clinical decision, while the result of that test represents patient state or physiology. For models that learn from clinician-initiated data and are expected to change clinical behavior, there should be an onus to demonstrate that the model is not merely looking over a clinician’s shoulder and quantifying a risk the clinician may already suspect. The latter operation is still valuable, as we elaborate below, but its performance critically depends on the presence and actions of clinicians up until the point that the machine learning model is applied.

An example of this distinction between clinician and non-clinician-initiated data can be seen in 3-year-survival differences between routine white blood cell counts and white blood cell counts taken during times unlikely to be routine. On average, patients with abnormal white blood cell counts have higher 3-year-survival rates than patients who have normal white blood cell counts taken at abnormal or nonroutine times^5^. Clinicians order specific panels of tests based on their clinical suspicion, expectations, or concerns about a patient’s state. If a test is ordered at an abnormal time it is likely to be nonroutine and may represent a concern on the part of the clinician. The primary difference between a blood test manually ordered in the middle of the night (clinician initiated) and a routine one (non-clinician initiated) is the decision-making agency of the physician. In the first case, a clinician chooses to order the test deliberately, based on concern prompted by examination of patient physiology. In contrast, when a test is part of a routine process, there is no selection on patient physiology or clinician expertise.

Finally, this feedback cycle between patient physiology and physician belief/action highlights the distinction between diagnostic and prognostic tasks, and the differing burdens of evidence and assumptions required for each. The underlying nature of a patient’s illness or condition is generally not dependent on the actions or beliefs of a physician. Consequently, the diagnostic process requires making a direct assessment of the patient’s physiology and condition. In contrast, prognosis involves making a prediction regarding the outcomes of a patient, and crucially assumes that the patient will receive a particular standard of care. Thus, a patient’s prognosis is tied to the specific actions undertaken by clinicians and is often dependent on the clinician-initiated act of diagnosis.

Predictions made from clinician-initiated actions may not accurately predict beyond what the average clinician would decide for the average similar patient. As an illustrative example, we can deconstruct the timing and frequency of actions and orders by a clinician for patient presenting to the emergency department with chest pain (Fig. 1b)^6^. In this example, a model utilizing this data may learn a test order for troponin means a patient is more likely to have a myocardial infarction (MI). Knowing that a patient has MI in combination with demographic risk factors and comorbidities may lead to impressive predictive performance for inhospital mortality but is unlikely to aid clinical decision making. This idea, that models are merely interpreting the existing thoughts of clinicians based on their actions rather than identifying true signal, may help explain why increased model performance has not translated to significant clinical impact in most applications of risk stratification^1,7^.

To evaluate the hypothesis that machine learning models may be modeling the existing reasoning of clinicians we quantified the ability of a deep neural network to predict patient outcomes using different subsets of data. We trained three models using: (1) patient demographic data only, (2) patient demographic data and data available at the time of presentation to the hospital, and (3) patient demographic data, data available at the time of presentation, and actions taken during the first day of admission. The performance of these models was compared to published state-of-the-art methods using complete EMR details.

## Results

### Description of data used and data generating processes

We compared prediction results using charge details to state-of-the-art benchmarks^8^ that utilize EMR-based clinical data, including notes, diagnoses, vital signs, histories, and laboratory orders/results. By evaluating the information content of a data source that contained exclusively clinician-initiated data elements, we could evaluate whether it was sufficient to achieve strong predictive performance on its own.

To do this, we utilized charge details, a data modality that represents a record of the specific tasks undertaken by a hospital for a specific patient and are used to help generate patient bills. These details represent the actions taken by clinicians (clinician-initiated data) and the resources used in order to provide care to a patient during a given encounter (Table 1, Supplementary Tables 1,2). However, because they are primarily an administrative product and not used for clinical decision making, they contain only the events that occurred, and resources used rather than physiological measurements of the patient. While it cannot be determined which individual actions or charges result from physician expertise, routine practice can be inferred by the frequency of charge patterns across the population (Table 1, Supplementary Tables 1, 2). The performance of the models provides evidence that it is able to differentiate between routine orders and orders based on clinical expertise.

**Table 1:** Example first day charge details for a patient with MI.

Description	Department	Quantity
EKG Routine tracing only	EKG	1
ECHO 2D W/OR W/O M-Mode complete W/color flow	Cardiology	1
ER Level V	Emergency room	1
XR Chest 2 views	Diagnostic imaging	1
Culture blood	Laboratory	2
Partial thromboplastin time (PTT)	Laboratory	1
Prothrombin time (PT)	Laboratory	1
Complete CBC AUTO W/O DIFF	Laboratory	1
TROPONIN QN	Laboratory	2
B-Type natriuretic peptide	Laboratory	1
Lactate/lactic acid	Laboratory	1
Creatine kinase (CPK) MB only	Laboratory	1
Creatine kinase (CPK)	Laboratory	2
Comprehensive metabolic panel	Laboratory	1
Therapeutic/DIAG INJ IV push single INITI SUB/drug	IV Therapy	1
DOCUSATE NA, COLACE CAP 100 mg	Pharmacy	1
Aspirin Tab 325 mg (EA)	Pharmacy	1
Moxifloxacin, Avelox IVPB 400 mg	Pharmacy	1
Moxifloxacin, Avelox tab 400 mg	Pharmacy	1
Metoprolol, lopressor tab 25 mg	Pharmacy	1
Ipratropium, atrovent INH SOL 0.02% 2.5 ml	Pharmacy	1
Heparin NA VL 5000 U/ml 1 ml	Pharmacy	1
Furosemide, Lasix tab 20 mg	Pharmacy	2
Albuterol, proventil INH SOL 0.083% 3 ml (2.5 mg)	Pharmacy	3
R&B Telemetry private	Room and board	1

Additionally, due to the de-identified nature of the data, timing and order of events within a day cannot be expected to be consistent or reliable. Importantly, because the 24 h period after admission cannot be identified, all predictions using charge data are done at the end of the first day of admissions and may include significantly <24 h of data.

Our analysis included 42,896,026 inpatient hospitalizations between 2013 and 2018 from 973 hospitals nationwide (Table 2, Supplementary Fig. 1). These hospitalizations included over 4.4 billion events occurring prior to and during the first day of admission as well as 21 static features available at the time of admission (demographic and provider details). In contrast, the EHR baseline of only 216,221 patients included more than 46.8 billion data points^8^. We constructed three sets of classifiers, based on (1) demographics only, (2) demographic and provider details only, and (3) demographic, provider, and charge details.

**Table 2:** Population information for data included for risk stratification using machine learning.

	2013	2014	2015	2016	2017	2018	Total
Hospitals included	778	783	797	786	770	755	973
Total encounters	79,209,178	82,145,811	85,037,615	85,391,057	84,448,480	84,641,611	500,873,752
Inpatient admissions	8,556,411	8,682,382	8,812,595	8,683,133	8,288,089	8,052,278	51,074,888
Multiday inpatient admissions	7,175,154	7,338,193	7,425,860	7,296,849	6,939,021	6,720,949	42,896,026
Total population: mortality	120,583 (1.68%)	123,764 (1.69%)	129,640 (1.75%)	126,844 (1.74%)	124,310 (1.79%)	121,549 (1.81%)	746,690 (1.74%)
Total population: extended length of stay	1,466,580 (20.44%)	1,492,958 (20.35%)	1,518,803 (20.45%)	1,506,125 (20.64%)	1,449,174 (20.88%)	1,437,552 (21.39%)	8,871,192 (20.68%)
Total population: 30-day readmission	941,911 (13.13%)	937,562 (12.78%)	950,561 (12.80%)	887,418 (12.16%)	925,833 (13.34%)	901,290 (13.41%)	5,544,575 (12.93%)
All MI admissions (% of all admissions)	69,448 (0.81%)	71,609 (0.82%)	78,975 (0.90%)	82,952 (0.96%)	84,407 (1.02%)	84,551 (1.05%)	471,942 (0.92%)
Multiday MI admissions (% of total multiday admissions)	56,594 (0.79%)	57,665 (0.79%)	63,026 (0.85%)	65,925 (0.90%)	66,859 (0.96%)	67,135 (1.00%)	319,539 (0.88%).
MI Cohort: mortality	3497 (6.18%)	3393 (5.88%)	3625 (5.75%)	3569 (5.41%)	3583 (5.36%)	3337 (4.97%)	21,004 (5.57%)
MI Cohort: extended length of stay	9172 (16.21%)	8941 (15.51%)	9463 (15.01%)	10,024 (15.21%)	10,036 (15.01%)	10,174 (15.15%)	57,810 (15.33%)

Due to the lack of event timing data, models trained with charge details were only given data up to the end of the first day of admission. In contrast, published benchmarks^8^ include full clinical details (including clinical notes) for the first 24 h after admission. Given that patients are admitted throughout the course of the day, many of the patients used to train our models had significantly <24 h of data which might handicap their performance.

To evaluate our hypothesis that clinical machine learning models based on a record of clinician-initiated actions are sufficient to predict inpatient outcomes, we constructed classifiers for three popular endpoints: mortality, readmission within 30 days, and extended length of stay (admissions of 7 days or more). We deployed these classifiers over all admissions lasting more than one day and included only the first day of a given stay in the classifier. Individual patients with more than one stay were classified separately, and no linkage between a given patient’s stays was created. Finally, the published EMR baselines performed resource intensive neural network architecture and hyperparameter searches for over 200,000 GPU h. The models trained on charges data were trained using basic architectures on two GPUs for all outcomes in <24 h.

### Comparison of model trained on clinician-initiated data only to published benchmarks

**Fig. 2:**
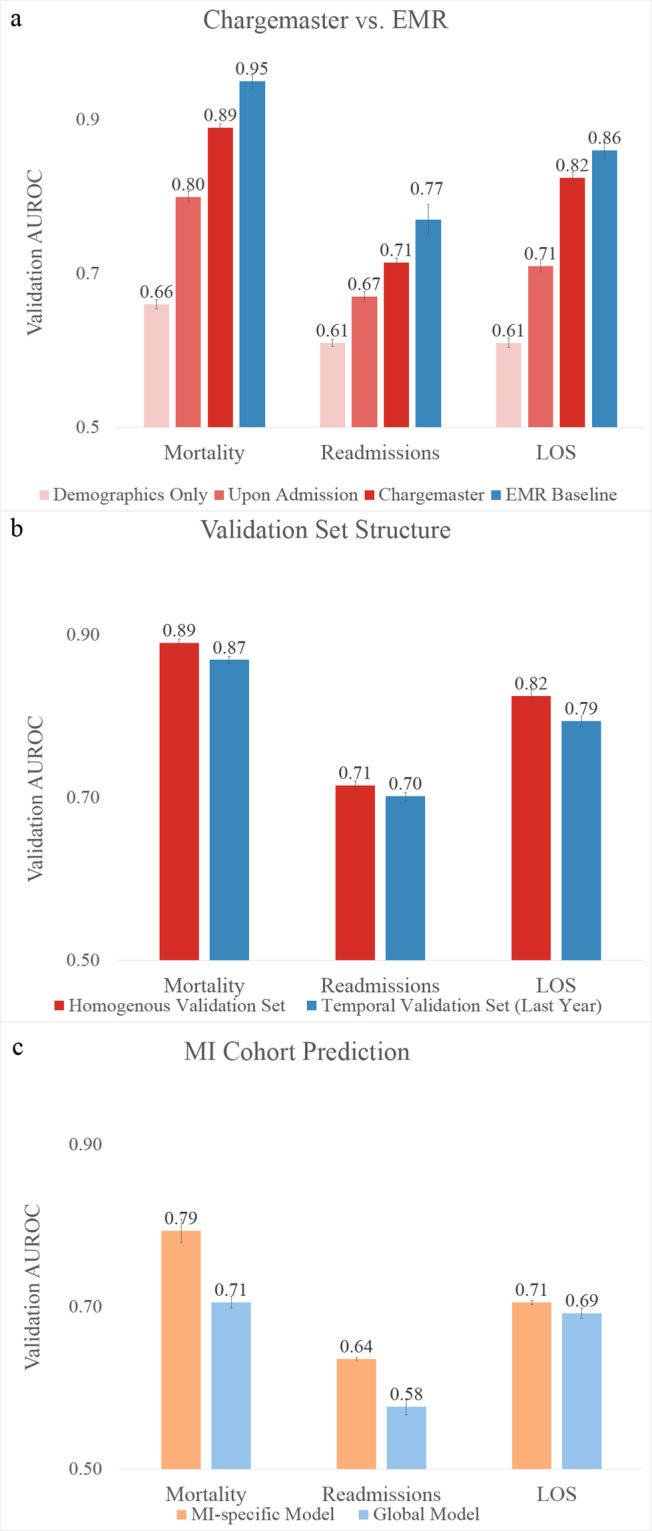
Performance comparison between charge and EMR data across cohorts and outcomes. **a** Comparison of mortality, readmission and length of stay performance (area under receiver-operating curve, AUROC) on randomly selected validation data. **b** Average relative features per patient for each model version. **c** Outcome comparison on a myocardial infarction (MI) patient cohort between models trained on MI patients exclusively and all available patients.

We found that relative to the published EMR baseline, abbreviated patient representations were able to capture significant amounts of signal for all three tasks (Fig. 2a). Charges data only modestly underperformed the baseline (AUCs of 0.89, 0.71, and 0.82 compared to 0.95, 0.77, and 0.86 for mortality, readmissions, and LOS respectively). Performance of the charges model was handicapped by the significant limitations intrinsic to fully de-identified charges data, including missing data modalities (e.g., laboratory values), significantly fewer total and per-patient data elements (Fig. 2b), lack of reliable event ordering (events were not necessarily in order through the day for de-identification), and the presence of data from only the day of admission (e.g., if a patient showed at 11:30 p.m. the model received only 30 min of data compared to a full 24 h in the benchmark). Direct comparison could not be made as exact times of service were limited to the day level. Additionally, the charges model was trained over a heterogeneous set of providers (*N* = 973) while the benchmark was fine tuned to two specific hospitals (we reported the highest results for the benchmark). Classifiers that utilized crude metrics of patient demographics and provider information captured the majority of signal relative to published EMR baselines over all three tasks. These results suggest that critical elements in EMR-based models are reflections and readouts of a clinician’s expertise.

However, clinical practice is highly sensitive to context, and the act of prognosis frequently involves implicit diagnostic prerequisites. Consequently, we hypothesized that models trained on clinician-initiated data would be better able to predict cohort-specific outcomes when patients outside the cohort (representing irrelevant patient presentations) were excluded. We trained a model specifically on patients who arrived at the emergency department suffering from MI. The MI cohort included MI patients hospitalized at hospitals with at least 100 such instances between 2013 and 2017. Models trained over this restricted subset demonstrated better performance predicting outcomes from MI hospitalizations in 2018 than the general model which was trained over all hospitalizations (Fig. 2c). The model trained with the more expansive training set underperformed relative to one trained on a targeted subset, thus emphasizing that the prognostic performance of these models is improved if clinicians identify a diagnosis with established standards of care. Divergence from this standard of care can then provide information to the model. Because these models derive signal primarily from patient interactions with healthcare providers, the observed effect may be caused by the potential for clinical actions to take on divergent interpretations when present in different contexts. The ability for a model trained generally to “guess” at a clinician’s thinking may be less effective when required to work across contexts, as the range of mechanisms that must be inferred is much wider.

## Discussion

The results of our experiments indicate machine learning models trained only on clinician-initiated administrative data can currently achieve performance close to models trained on more detailed, complete, EMR data. This is an important result because it provides insight into the current utility of machine learning models for patient risk stratification from clinical data and the primary source of signal that these models utilize. This indicates that current models extrapolate from the thinking of the clinician manifested through their clinical actions, and that strong prognostic performance may only represent the diagnostic value of the clinician-initiated data points they rely on. The results of our experiments also indicate the value of easier to access, lower resolution datasets (e.g., administrative vs. EMR). Finally, the results provide baseline performance levels that should be exceeded prior to claims that machine learning models can provide tangible guidance to clinicians, rather than simply looking over their shoulders.

Given the relative performance between current models with clinician-initiated and non-clinician-initiated data, it will be important to show that performance improvements are the result of non-clinician-initiated data (e.g., raw imaging results) or data that are difficult or expensive to interpret (e.g., constant real time streaming data). Without this demonstration we should be cautious about assuming predictive models can improve individual-level decisions. In fact, if model performance is driven by the actions of the clinician (e.g., a test order) and not the underlying physiology (e.g., a test result) the model has the potential to confuse a clinician. For example, if a clinician orders a test because they suspect a condition with poor prognosis, but that test comes back normal, the clinician may rule out the condition, but the model may state the patient is at high risk based on the clinician’s original test order. The model is unlikely to be accurate until it observes the next steps taken by the clinician. However, if a clinician sees the patient is still at high risk, they may take actions that differ from their normal standard of care to try to determine why the model is stating the patient is at high risk. This change in behavior may induce dataset shift, by causing a difference between the retrospective data the model was trained on and the prospective data it is applied to.

The idealized use case of machine learning models for patient risk stratification is to have generalizable models that provide specific and personalized projections for individual patients. However, models that derive their predictions from clinician-initiated data may produce predictions based on what a physician would do for an average, similarly presenting patient, rather than the individual patient in question. Acknowledging the selective role that clinicians play in terms of what decisions and actions they choose to make on what data is available for models is critical for developing models that can truly assist clinician decision making. Acknowledging this role also points to a potentially important application of models trained on clinician-initiated data: recognizing where clinical decision making diverges from machine learning models trained directly on patient state or physiological data. For example, if the clinician-initiated model predicts a patient is low risk based on the actions a physician takes after a CT pulmonary angiography, but a model trained specifically to read CT pulmonary angiograms predicts the patient that is at high risk, it can be said that the physician’s decisions and the CT image model have diverged. This is an indication that either the model is misreading the CT image, or the physician has not recognized something of danger. Divergence might occur if a hyperdense lesion exists within the pulmonary arterioles, but the physician can’t tell from the imaging and believes it exists within the bronchial tree. This would lead the physician’s diagnosis away from pulmonary embolism. Regardless of whether the physician or the CT model had been right, the divergence would warrant further investigation to either prevent the misdiagnosis of a patient or to update and correct the CT model.

While models may superficially display strong prognostic performance, if this performance is derived from the diagnostic efforts of physicians, the model cannot truly be relied upon as if it were acting independently. The true physiological state of a patient is often incompletely characterized and obfuscated through various sources of bias in the electronic medical record (EMR)^9–13^. Despite this, most current machine learning investigations utilizing these data rely on a major simplifying assumption: that the state of a patient can be inferred through the use of routinely collected data in the EMR^14^. These data encode information about how clinicians and the healthcare system as a whole reacted react to the patient, potentially confounding prediction models built to use it. Machine learning models trained using EMR-derived features are consequently linked to the individual decisions and assessments made by clinicians. This observation can also explain the necessity for models to be retrained across institutions. The physiological phenomena underpinning disease are largely static, but physicians have diverse behavior profiles corresponding to different disease trajectories that might not be captured in a single training set.

Freed from the expense of collecting clinical details and the epistemological burden of predicting individual patient outcomes in an unbiased manner, current risk stratification models could have tremendous utility in allowing patients to view quantified prognoses, as well as guiding value-based care decisions, hospital logistics and staffing management. This is especially true using administrative byproducts such as charges details. Acknowledging these models are effective at learning clinician’s prognoses through their actions, rather than attempting to assist with individual decisions, these models could be used to quantify cohort or population level prognoses. An example would be a tool providing administrators with a more holistic view for the current inpatient load and acuity levels of their patients. Such a tool could enable better planning, staffing and resource allocation. The ability to train cohort-specific models also suggest values in lower resolution administrative datasets which may have larger patient counts that allow for the training specialized models. A key challenge in this endeavor will be to identify cohorts prospectively to choose which model should be used.

The promise of machine learning in healthcare necessitates an understanding of where the dominant sources of predictive signal are located, as well as what information is truly useful in shifting marginal decisions. Through an understanding of the unique conditions in which healthcare data are created and utilized, researchers can better identify the cases where machine predictions are likely to be beneficial.

## Methods

### Data

The Premier Healthcare Database (PHD)^15^ is a large-scale, provider-based, all-payer database containing data on more than 215 million total patients and 115 million inpatient admissions. It includes more than six million inpatient admissions each year between 2013 and 2018 and a total of over 35 million admissions more than one day between 2013 and 2017 (training) and 6.7 million admissions lasting longer than one day in 2018 (test) (Table 2). The PHD has been certified as de-identified via expert determination in compliance with HIPAA. The research was deemed to be “nonhuman” in consultation with the Harvard Medical School IRB.

The PHD contains information on providers (hospital, organizational and clinician) and visit characteristics. It includes patient demographics, disposition and discharge information as well as diagnoses for admission and discharge, and billed services such as procedure orders, medication and device orders, laboratory test orders, as well as diagnostic and therapeutic services. The PHD is an administrative database for quality improvement and does not include laboratory test results, vital signs, patient notes or other data modalities that are commonly available in EMRs.

#### Subsets of data

1. Demographic data only:
  Age, gender, race, marital status, and type of insurance (e.g., private, public, government etc.).
2. Demographic data and information available at time of admission:
  All from #1 and admission month, source of admission (e.g., another healthcare provider, home etc.), type of admission (e.g., emergency, urgent, elective), admitting physician specialty, point of origin (e.g., emergency department, obstetrics and gynecology etc.).
3. Demographic data, information at admission and all charges during the first calendar day of admission:
  All from #1 and #2 as well as charge codes for all actions taken from presentation at the hospital until the end of the first calendar day of admission.

### Cohort selection

Both cohorts and the timing of the prediction were selected to match the structure of those of the baseline^14^. We include predictions of inpatient mortality, 30-day readmission, prolonged length of stay (>7 days). All available hospitalizations with length of stay greater than 1 day were included, and separate hospitalizations of the same patient were treated separately. Hospitalizations that ended in mortality were excluded from cohorts predicting readmission, and hospitalizations that ended in mortality after <7 days were excluded from cohorts predicting prolonged length of stay. For the general classifiers, all hospitalizations were used, while for the MI classifiers, those with an MS-DRG corresponding to “acute myocardial infarction” (AMI) were selected (280–285). AMI was selected for its higher-than-average mortality rate and low presentation to diagnosis time in general.

### Model architecture and training

To make these predictions we first learn 8-dimensional clinical concept embeddings as in Beaulieu-Jones et al.^16^ for 36,089 distinct charges using 94,708,714 co-occurrence pairs and 146,531,783,286 total relationships. Charges over the first day are converted into a sequence 100 events long and pre-padding with 0’s and pre-clipping where necessary.

Two separate model architectures were utilized depending on the type of data utilized: models based on demographics and provider details utilized logistic regression due to the small number of features, while those based on charge data utilized a stacked recurrent neural network (gated recurrent unit (GRU)). Models were trained using the Adam optimizer^17^ until convergence based on validation accuracy-informed early stopping. Dropout regularization was applied to each model. A table of model hyperparameters is provided in Supplementary Table 7. Data preprocessing was done using Spark^18,19^ via Pyspark to query a high performance Hadoop cluster. Preprocessed data was saved in parquet format and fed to models in the TensorFlow framework^20^ using Petastorm^21^. All models were run on edge nodes with 72 CPU cores and four Nvidia V100 GPUs. Source code to preprocess the data and train example models with random validation cohorts is available on GitHub (https://github.com/brettbj/inpatient-stratification-charges) and archived on Figshare^22^. Parameter selection, training and evaluation were all designed to prevent any chance of overfitting or any claim of architectural superiority as the cause of relative performance. Because of this, we minimized the parameter sweep (as demonstrated by round, commonly chosen numbers) and used orders of magnitude less compute to train our models. All of our models train on four GPUs in <24 h, in comparison to >201,000 total GPU hours for the benchmark (compute statistics available in preprint only)^23^. Additionally, to avoid any impression about overfitting, our example trains one model for each outcome across all 973 health systems and does not perform fine-tuning, where the benchmark tunes for each health system.

### Evaluation

Models were randomly partitioned into training, validation, and test sets in an 80:10:10 ratio respectively. Area under the receiver-operating curve was the primary metric for evaluating and comparing model performance. Models were selected by comparing performance on the validation set and then evaluated on the test set.

### Reporting summary

Further information on research design is available in the Nature Research Reporting Summary linked to this article.

## Supplementary information

Supplementary Information

Reporting Summary

## Data Availability

All data used in this study are from the PHD^15^. These data may be purchased from Premier Inc., https://www.premierinc.com/solutions/applied-sciences. Code for deriving training, validation, and test datasets is available on GitHub and authors can provide confirmatory de-identified record IDs for each set upon reasonable request.

## Code availability

All source code used in this study are available on GitHub (https://github.com/brettbj/inpatient-stratification-charges) and in archival form on Figshare^22^. Analyses were preformed using Python (3.6.8), Scikit-Learn (0.22), Apache Spark (2.4.0), and Tensorflow (1.13.1).
