# Supplementary material for: Machine learning for patient risk stratification: standing on, or looking over, the shoulders of clinicians?
Source: NPJ Digit Med. 2021 Mar 30;4:62. doi: 10.1038/s41746-021-00426-3 (PMC8010071; doi:10.1038/s41746-021-00426-3)
Supplement: Supplementary file 1 — Supplementary Information [file 41746_2021_426_MOESM1_ESM.pdf]

Supplementary Materials

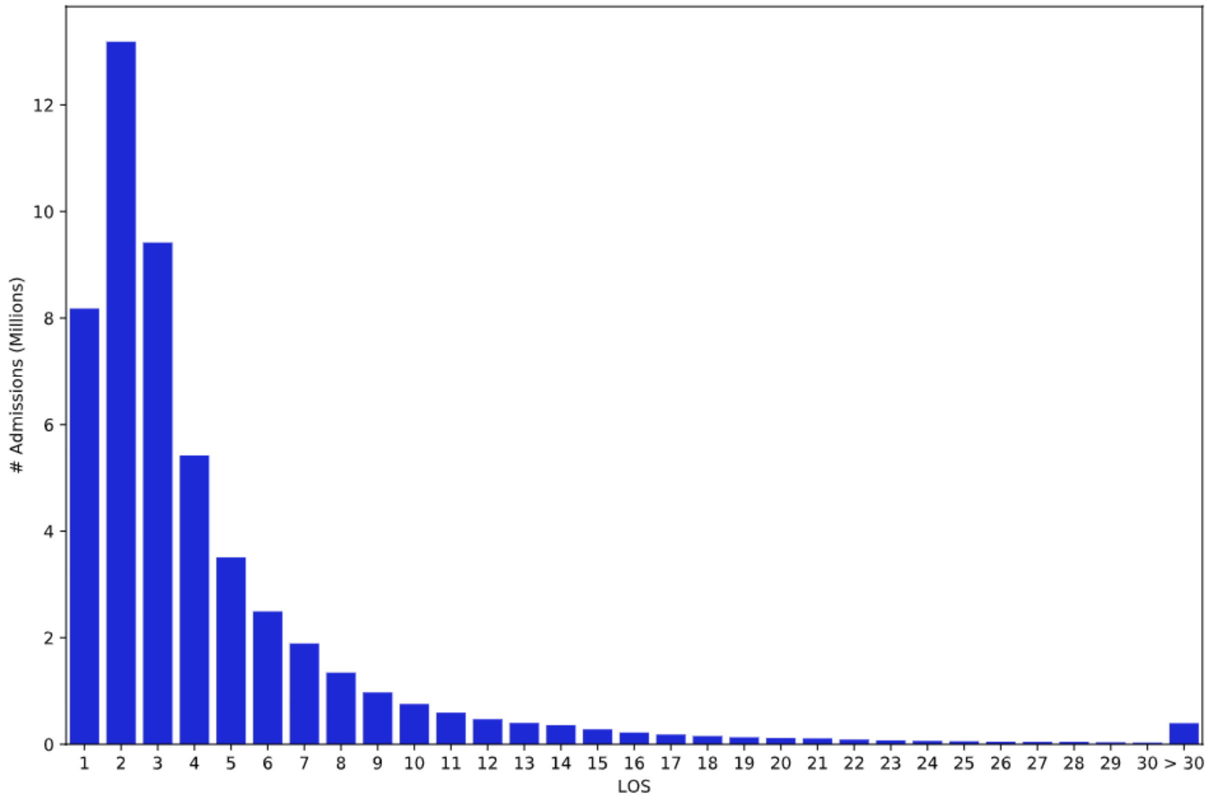

Supplementary Figure 1. Histogram of length of stays for included admissions (in millions).

Supplementary Table 1. Example first day charges for an MI patient with little data available.

Description	Department	Quantity
PF ER LEVEL V	PROFESSIONAL FEES	1
EKG ROUTINE TRACING ONLY	EKG	2
ER LEVEL V	EMERGENCY ROOM	1
THERAPEUTIC/DIAG INJ IV PUSH SINGLE INITI SUB/DRUG	IV THERAPY	1
*LORAZEPAM, ATIVAN INJ 2MG	PHARMACY	1
R&B ONCOLOGY PRIVATE	ROOM AND BOARD	1

Supplementary Table 2. Example first day charges with unclear treatment actions.

Description	Department	Quantity
PF ER LEVEL V	PROFESSIONAL FEES	1
EKG ROUTINE TRACING ONLY	EKG	2
PRESSURIZED OR NONPRESSURIZED INHALATION TX	RESPIRATORY THERAPY	1
ER LEVEL V	EMERGENCY ROOM	1
CPAP PER DAY	RESPIRATORY THERAPY	1
CPAP PER DAY	RESPIRATORY THERAPY	1
RT TIME FLAT RATE	RESPIRATORY THERAPY	1
*XR CHEST 1 VIEW PORTABLE	DIAGNOSTIC IMAGING	1
CULTURE BLOOD	LABORATORY	2
PARTIAL THROMBOPLASTIN TIME (PTT)	LABORATORY	1
PROTHROMBIN TIME (PT)	LABORATORY	1
COMPLETE CBC AUTO W/AUTO DIFF	LABORATORY	1
TROPONIN QN	LABORATORY	1
LACTATE/LACTIC ACID	LABORATORY	1
GLUCOSE BY DEVICE	LABORATORY	1
CREATINE KINASE (CPK) MB ONLY	LABORATORY	1
CREATINE KINASE (CPK)	LABORATORY	1
COMPREHENSIVE METABOLIC PANEL 80053	LABORATORY	1
NEW THERA PROPHY/DIAG INJ EA ADD SEQ PUSH SUB/DRUG	IV THERAPY	2
IV INFUSION SUBSTANCES/DRUG CONCURRENT 96368	IV THERAPY	1
IV INFUSION SUBST/DRUG EA ADD SEQ UP TO 1 HR 96367	IV THERAPY	1
IV INFUSION SUBSTANCES/DRUGS EACH ADDL 1 HR 96366	IV THERAPY	3
IV INFUSION SUBSTANCES/DRUGS UP TO 1 HR 96365	IV THERAPY	1
0.9% NACL VL 10ML	PHARMACY	1
0.9% NACL 250ML	PHARMACY	1
ASPIRIN TAB CHW 81MG (EA)	PHARMACY	4
ALBUTEROL, NONCOMP INH SOL 1MG	PHARMACY	5

INSULIN ASPART PROT/ASPART, NOVOLOG 70/30 PER DOSE	PHARMACY	0.05
IPRATROPIUM/ALBUTEROL, DUONEB INH SOL 3ML	PHARMACY	1
LIDOCAINE, XYLOCAINE VL 2% 10ML	PHARMACY	1
HEPARIN NA VL 1,000U/ML 1ML	PHARMACY	1
HEPARIN NA VL 1,000U/ML 1ML	PHARMACY	1
*FUROSEMIDE, LASIX VL 40MG 4ML	PHARMACY	3
FENTANYL, SUBLIMAZE AMP 0.05MG/ML 2ML	PHARMACY	1
CEFTRIAXONE, ROCEPHIN VL 250MG	PHARMACY	1
AZITHROMYCIN, ZITHROMAX VL 500MG	PHARMACY	1
NON REVENUE ITEM	ADMINISTRATIVE FEES	5
R&B ICU	ROOM AND BOARD	1

Supplementary Table 3. Admitting Physician Specialty (where available).

Admitting Physician Specialty	# Admissions
INTERNAL MEDICINE (IM)	8,683,840
HOSPITALIST (HOS)	7,668,355
OBSTETRICS/GYNECOLOGY (OBG)	4,295,512
UNKNOWN	3,254,355
PEDIATRICS (PD)	3,209,914
FAMILY PRACTICE (FP)	2,286,362
ORTHOPEDIC SURGERY (ORS)	1,824,100
PSYCHIATRY (P)	1,765,802
GENERAL SURGERY (GS)	1,479,856
NEONATAL - PERINATAL MEDICINE (NPM)	892,949
CARDIOVASCULAR DISEASES (CD)	876,613
UNSPECIFIED (US)	672,255
NEUROLOGICAL SURGERY (NS)	433,030
PULMONARY DISEASES (PUD)	426,366
PHYSICAL MEDICINE AND REHAB (PM)	348,622
EMERGENCY MEDICINE (EM)	331,833
OTHER SPECIALTY (OS)	297,251
CRITICAL CARE MEDICINE (CCM)	255,352
NEPHROLOGY (NEP)	235,647
UROLOGY (U)	226,721
THORACIC SURGERY (TS)	225,508
CARDIOVASCULAR SURGERY (CDS)	219,539
HEMATOLOGY/ONCOLOGY (HO)	199,818
VASCULAR SURGERY (VS)	198,145
TRAUMA SURGERY (TRS)	171,277
NEUROLOGY (N)	170,404
OBSTETRICS (OBS)	141,561
COLON/RECTAL SURGERY (CRS)	141,120
PULMONARY CRITICAL CARE MEDICINE (PCC)	100,151

PEDIATRIC CRITICAL CARE MEDICINE (CCP)	92,561
GYNECOLOGICAL ONCOLOGY (GO)	84,902
CERTIFIED NURSE MIDWIFE (CNM)	80,432
MEDICAL ONCOLOGY (ON)	79,390
INTERVENTIONAL CARDIOLOGY	73,798
GASTROENTEROLOGY (GE)	73,657
MATERNAL AND FETAL MEDICINE (MFM)	73,394
CHILD AND ADOLESCENT PSYCHIATRY (CHP)	72,002
GERIATRICS - INTERNAL MEDICINE (IMG)	63,311
GENERAL PRACTICE (GP)	63,155
SURGICAL CRITICAL CARE (CCS)	62,820
INTENSIVIST (INT)	62,410
INFECTIOUS DISEASES (ID)	61,761
GYNECOLOGY (GYN)	60,553
PEDIATRIC HEMATOLOGY/ONCOLOGY (PHO)	57,319
PLASTIC SURGERY (PS)	52,931
PEDIATRIC SURGERY (PDS)	47,671
OTOLARYNGOLOGY (OTO)	46,239
NURSE PRACTITIONER (ARNP)	45,990
ORTHOPEDIC SURGERY OF THE SPINE (OSS)	40,801
SURGICAL ONCOLOGY (SO)	35,813
CARDIAC ELECTROPHYSIOLOGY (ICE)	34,263
ANESTHESIOLOGY (AN)	33,296
PODIATRY (POD)	27,802
ENDOCRINOLOGY AND METABOLISM (END)	27,738
GERIATRIC MEDICINE - FAMILY PRAC. (FPG)	26,737
HEMATOLOGY (HEM)	24,599
TRANSPLANT SURGERY (TTS)	22,886
SPORTS MEDICINE - ORTHOPEDICS (OSM)	17,005
PHYSICIAN ASSISTANT (DRA)	15,025
PEDIATRIC GASTROENTEROLOGY (PG)	14,909

HEMATOLOGY (HMP)	14,270
RHEUMATOLOGY (RHU)	12,860
DENTAL/ORAL SURGERY (DOR)	12,790
PEDIATRIC PULMONOLOGY (PDP)	12,645
ADDICTION MEDICINE (ADM)	11,677
ADOLESCENT MEDICINE (ADL)	11,538
PEDIATRIC NEPHROLOGY (PN)	11,536
PEDIATRIC INFECTIOUS DISEASES (PDI)	11,382
PEDIATRIC EMERGENCY MEDICINE (PEM)	11,054
PEDIATRIC CARDIOLOGY (PDC)	10,281
HAND SURGERY (HS)	10,273
PSYCHOANALYSIS (PYA)	9,921
PAIN MANAGEMENT (APM)	9,236
PEDIATRIC NEUROLOGY (CHN)	8,820
VASC. & INTERVENTIONAL RADIOLOGY (VIR)	8,050
HOSPICE & PALLIATIVE CARE	7,694
RADIOLOGY - DIAGNOSTIC (DR)	7,337
CERTIFIED REG. NURSE ANESTHETIST (CRNA)	7,067
RADIOLOGY (R)	6,868
ABDOMINAL SURGERY (AS)	6,725
RADIATION ONCOLOGY (RO)	6,093
PEDIATRIC ORTHOPEDICS (OP)	5,976
OPHTHALMOLOGY (OPH)	5,900
PEDIATRIC SURGERY - NEUROLOGICAL (NSP)	5,247
ALLERGY AND IMMUNOLOGY (AI)	4,745
MEDICAL GENETICS (MG)	4,503
PEDIATRIC ALLERGY (PDA)	4,327
NUCLEAR MEDICINE (NM)	4116
REPRODUCTIVE ENDOCRINOLOGY (REN)	4,003
DERMATOLOGY (D)	3,986
SPORTS MEDICINE (FSM)	3,540

PEDIATRIC ENDOCRINOLOGY (PDE)	3,336
PEDIATRIC UROLOGY (UP)	2,557
SLEEP MEDICINE	2,533
OCCUPATIONAL MEDICINE (OM)	2,460
MAXILLOFACIAL SURGERY	2266
ALLERGY (A)	2,115
ANATOMIC/CLINICAL PATHOLOGY (PTH)	2,000
SPORTS & INTERNAL MEDICINE (ISM)	1,413
GENERAL PREVENTATIVE MEDICINE (GPM)	1,254
NEUROPATHOLOGY (NP)	1,237
OSTEOPATHIC MANIPULATIVE MEDICINE (OMM)	1,210
CLINICAL GENETICS (CG)	1,149
LEGAL MEDICINE (LM)	1,072
PEDIATRIC OTOLARYNGOLOGY (PDO)	1,046
HEAD AND NECK SURGERY (HNS)	1,017
CLINICAL PHARMACOLOGY (PA)	905
CHIROPRACTICE (CRP)	780
FACIAL PLASTIC SURGERY (FPS)	744
NUCLEAR RADIOLOGY (NR)	579
DIABETES (DIA)	565
NEURORADIOLOGY (RNR)	522
PUBLIC HLTH & GEN?L PREV. MEDICINE (PHP)	508
DERMATOPATHOLOGY (DMP)	460
NUTRITION (NTR)	458
CLINICAL PATHOLOGY (CLP)	388
PSYCHOLOGIST, CLINICAL	256
CLINICAL NEUROPHYSIOLOGY (CN)	230
PEDIATRIC OPHTHALMOLOGY (PO)	220
PEDIATRIC RHEUMATOLOGY (PPR)	200
CERTIFIED CLINICAL NURSE SPECIALIST	174
ANATOMIC PATHOLOGY (ATP)	146

IMMUNOLOGY (IG)	146
CHEMICAL PATHOLOGY (PCH)	125
PHYSICAL THERAPY	18
OCCUPATIONAL THERAPY	14
PEDIATRIC RADIOLOGY (PDR)	14
OPTOMETRY	10

Supplementary Table 4. Admission Type (where available).

Admission Type	# Admissions
EMERGENCY	22,237,533
ELECTIVE	8,868,840
URGENT	6,657,028
NEWBORN	4,352,538
TRAUMA CENTER	357,779

Supplementary Table 5. Major Diagnostic Category Assigned.

Major Diagnostic Category	Category Name	# Admission
5	DISEASES/DISORDER/CIRC SYSTEM	5,316,778
14	PREG/CHILDBIRTH/PUERPERIUM	5,128,548
15	NEWBORNS/OTHER NEONATES	4,555,578
4	DISEASES/DISORDER/RESP SYSTEM	4,027,823
8	DISEASES/DISORDER/MS SYSTEM	3,927,870
6	DISEASES/DISORDER/DIGES SYSTEM	3,587,412
18	INFECTIOUS AND PARASITIC DISEA	2,864,044
1	DISEASES/DISORDER/NERV SYSTEM	2,514,318
11	DISEASES/DISORDER/KIDNEY/UT	2,039,612
19	MENTAL DISEASES AND DISORDERS	1,978,047
10	DISEASES/DISORDER/ENDOC/METAB	1,295,756
7	DISEASE/DISORDER/HEPATOBI/PANC	1,279,618
9	DISEASES/DISORDER/SKIN/SQ TISS	969,806
21	INJ/POISONING/TOXIC EFFECTS/DR	563,563
20	ALCOHOL/DRUG USE/INDUCED MENTA	530,731
23	FACTORS INFLUENC HEALTH STATUS	529,450
16	DISEASE/DISORDER/BLD/BLD FORMI	498,960
13	DISEASES/DISORDER/FEMALE REP	352,369

17	DISEASE/DISORDER/MYELOPROLIFER	296,020
3	DISEAS/DISORDER/EAR,NOSE,MOUT	292,849
12	DISEASES/DISORDER/MALE REP SYS	117,478
24	MULTIPLE SIGNIFICANT TRAUMA	110,990
25	HIV INFECTIONS	59,706
2	DISEASES/DISORDER/EYE	38,184
22	BURNS	18,806

Supplementary Table 6. Medicare Severity-Diagnosis Related Group (MS-DRG) Assigned.

MS-DRG	MS-DRG Description	# Admissions
795	NORMAL NEWBORN	2,776,356
775	VAGINAL DELIVERY W/O COMPLICATING DX	2,430,494
885	PSYCHOSES	1,562,790
871	SEPTICEMIA/SEVR SEPSIS W/OMV >96HRS WMCC	1,485,867
470	MJR JNT RPLCMNT/RTTHMNT OF LWR ET W/OMCC	1,302,965
766	CESAREAN SECTION W/O CC/MCC	1,000,358
794	NEONATE W OTHER SIGNIFICANT PROBLEMS	987,182
291	HEART FAILURE & SHOCK W MCC	716,856
765	CESAREAN SECTION W CC/MCC	677,839
392	ESOPHAGITIS, GI&MSC DIGST DISORDR W/OMCC	675,592
872	SEPTICEMIA/SEVR SEPSS W/OMV >96HRSW/OMCC	570,280
603	CELLULITIS W/O MCC	525,893
189	PULMONARY EDEMA & RESPIRATORY FAILURE	445,481
774	VAGINAL DELIVERY W COMPLICATING DX	433,856
683	RENAL FAILURE W CC	412,598
190	CHRONIC OBSTRUCTIVE PULMNRY DISEASE WMCC	400,233
897	ALC/DRUG ABUSE OR DEPND W/O REHAB W/OMCC	399,245
690	KIDNEY & URINARY TRACT INFECTIONS W/OMCC	397,738
292	HEART FAILURE & SHOCK W CC	396,130
193	SIMPLE PNEUMONIA & PLEURISY W MCC	391,168

194	SIMPLE PNEUMONIA & PLEURISY W CC	386,348
378	G.I. HEMORRHAGE W CC	384,657
247	PERC CRDVSC PX W DRUG-ELUT STENT W/O MCC	323,993
65	INTRACRNIAL HEM OR CEREBRAL INFARCT WCC	317,635
682	RENAL FAILURE W MCC	316,965
853	INFECTIOUS & PARASITIC DIS W OR PX WMCC	274,883
641	MISC DISORD NUTR,METABL,FLD/ELCTR WO MCC	272,363
793	FULL TERM NEONATE W MAJOR PROBLEMS	272,253
191	CHRONIC OBSTRUCTIVE PULMONRY DISEASE WCC	262,319
460	SPINAL FUSION EXCEPT CERVICAL W/O MCC	254,288
638	DIABETES W CC	245,239
945	REHABILITATION W CC/MCC	238,268
792	PREMATURITY W/O MAJOR PROBLEMS	236,535
330	MAJOR SMALL & LARGE BOWEL PX W CC	235,441
287	CIRC DISORDRS EXC AMI, W CRD CATH W/OMCC	224,784
309	CARD ARRHYTHMIA & CONDUCT DISORDERS WCC	224,382
812	RED BLOOD CELL DISORDERS W/O MCC	219,580
64	INTRACRNIAL HEM OR CEREBRAL INFARCT WMCC	218,146
481	HIP & FEMUR PX EXCEPT MAJOR JOINT W CC	213,091
177	RESPIRATORY INFECT & INFLAMMATIONS W MCC	193,413
101	SEIZURES W/O MCC	190,518
689	KIDNEY & URINARY TRACT INFECTIONS W MCC	189,943
208	RESPIRATORY SYS DX W VENT SUPPORT <=96 H	185,556
280	ACUTE MI, DISCHARGED ALIVE W MCC	184,516
881	DEPRESSIVE NEUROSES	183,397
308	CARD ARRHYTHMIA & CONDUCT DISORDERS WMCC	181,750
312	SYNCOPE & COLLAPSE	181,620
202	BRONCHITIS & ASTHMA W CC/MCC	181,284
640	MISC DISORD NUTR,METABL,FLD/ELCTRL W MCC	166,288
310	CARD ARRHYTH&CONDUCT DISORDERS W/OCC/MCC	162,312
439	DISORDER OF PANCREAS EXC MALIGNANCY W CC	161,339

917	POISONING & TOXIC EFFECTS OF DRUGS W MCC	160,630
377	G.I. HEMORRHAGE W MCC	160,629
552	MEDICAL BACK PROBLEMS W/O MCC	159,816
195	SIMPLE PNEUMONIA & PLEURISY W/O CC/MCC	150,440
389	G.I. OBSTRUCTION W CC	150,340
781	OTH ANTEPARTUM DX W MEDICAL COMPLICATION	148,762
203	BRONCHITIS & ASTHMA W/O CC/MCC	144,612
57	DEGENERATIV NRVOUS SYSTM DISORDRS W/OMCC	142,676
743	UTERINE&ADNEXA PX NONMALIGNACY W/OCC/MCC	138,044
192	CHRONIC OBSTRUCTV PULM DISEASE W/OCC/MCC	135,093
331	MAJOR SMALL & LARGE BOWEL PX W/O CC/MCC	134,553
918	POISONING&TOXIC EFFECTS OF DRUGS W/O MCC	133,184
329	MAJOR SMALL & LARGE BOWEL PX W MCC	132,173
394	OTHER DIGESTIVE SYSTEM DIAGNOSES W CC	130,935
313	CHEST PAIN	130,507
419	LAPSCP CHOLECYSTECTOMY W/O CDE W/OCC/MCC	130,491
246	PRC CRDVSCPX W DRGELT ST WMCC OR 4+VS/ST	129,482
391	ESOPHAGITIS, GI & MSC DIGST DISORDR WMCC	128,907
698	OTHER KIDNEY & URINARY TRACT DX W MCC	128,121
176	PULMONARY EMBOLISM W/O MCC	127,999
790	EXTREME IMMATURITY OR RDS, NEONATE	126,748
66	INTRCRNIAL HEM/CEREBRL INFARCT W/OCC/MCC	125,511
314	OTHER CIRCULATORY SYSTEM DIAGNOSES W MCC	124,071
791	PREMATURITY W MAJOR PROBLEMS	123,055
390	G.I. OBSTRUCTION W/O CC/MCC	118,589
418	LAPRSCOPIC CHOLECYSTECTOMY W/O CDE W CC	118,502
69	TRANSIENT ISCHEMIA	115,630
281	ACUTE MI, DISCHARGED ALIVE W CC	114,445
621	O.R. PROCEDURES FOR OBESITY W/O CC/MCC	112,080
293	HEART FAILURE & SHOCK W/O CC/MCC	111,076
440	DISORDER OF PANCREAS EXC MALIG W/OCC/MCC	110,675

948	SIGNS & SYMPTOMS W/O MCC	110,119
286	CIRC DISORDERS EXC AMI, W CARD CATH WMCC	105,596
637	DIABETES W MCC	105,462
372	MJR GI DISORDRS & PERITONEAL INFECT WCC	104,567
870	SEPTICEMIA OR SEVERE SEPSIS WMV >96 HOUR	102,306
300	PERIPHERAL VASCULAR DISORDERS W CC	96,469
253	OTHER VASCULAR PROCEDURES W CC	95,693
178	RESPIRATORY INFECT & INFLAMMATIONS W CC	95,088
639	DIABETES W/O CC/MCC	94,300
981	EXT OR PX UNRELATED TO PRINCIPAL DX WMCC	94,231
854	INFECTIOUS & PARASITIC DIS W OR PX W CC	93,773
100	SEIZURES W MCC	89,986
494	LWR EXT&HMR PX EXC HIP,FT,FMR W/OCC/MCC	89,401
699	OTHER KIDNEY & URINARY TRACT DX W CC	85,864
252	OTHER VASCULAR PROCEDURES W MCC	85,026
847	CHEMO W/O ACUTE LEUKEMIA SECONDR DX W CC	83,729
807	VAG DELIV W/O STERILIZATION/D&C W/O CC/M	83,591
236	CORONARY BYPASS W/O CARDIAC CATH W/O MCC	83,155
207	RESPIRATORY SYS DX W VENT SUPPORT >96HRS	82,906
305	HYPERTENSION W/O MCC	82,707
493	LWR EXT&HUMER PX EXC HIP,FOOT,FEMUR W CC	82,047
25	CRANIOTOMY&ENDOVASC INTRACRANIAL PX WMCC	80,141
767	VAGINAL DELIVERY W STERILIZATION&/OR D&C	79,522
74	CRANIAL&PERIPHERAL NERVE DISORDRS W/OMCC	79,320
175	PULMONARY EMBOLISM W MCC	79,049
234	CORONARY BYPASS W CARDIAC CATH W/O MCC	78,532
602	CELLULITIS W MCC	77,625
811	RED BLOOD CELL DISORDERS W MCC	77,568
442	DSRDRS LIVER EXC MALG,CIRR,ALC HEPA W CC	77,397
482	HIP & FEMUR PX EXC MAJOR JOINT W/OCC/MCC	77,162
884	ORGANIC DISTURBANCES&MENTAL RETARDATION	75,048

480	HIP & FEMUR PX EXCEPT MAJOR JOINT W MCC	74,681
166	OTHER RESP SYSTEM O.R. PROCEDURES W MCC	73,721
432	CIRRHOSIS & ALCOHOLIC HEPATITIS W MCC	72,090
441	DSRDRS LIVER EXC MALG,CIRR,ALC HEPA WMCC	72,011
153	OTITIS MEDIA & URI W/O MCC	70,070
417	LAPRSCOPIC CHOLECYSTECTOMY W/O CDE W MCC	69,529
469	MJR JNT RPLCMNT/RTTHMNT OF LWR EXT WMCC	69,431
3	ECMO/TRCHWMV>96HR/PDXEXFCE/MTH&NCKW/MJOR	68,953
220	CRD VLV&OTH MJ CD/THR PX W/O CRD CTH WCC	68,430
742	UTERINE&ADNEXA PX NONMALIGNANCY WCC/MCC	68,060
103	HEADACHES W/O MCC	67,593
393	OTHER DIGESTIVE SYSTEM DIAGNOSES W MCC	66,746
92	OTHER DISORDERS OF NERVOUS SYSTEM W CC	65,785
243	PERMANENT CARDIAC PACEMAKER IMPLANT W CC	65,634
809	MJR HEM/IMM DX EXC SCKL CL CRS&COAG W CC	65,341
563	FX,SPN,STN&DSL EX FMR,HP,PLVS&TGH W/OMCC	63,408
467	REVISION OF HIP OR KNEE REPLACEMENT W CC	63,360
438	DISORDER OF PANCREAS EXC MALIGNANCY WMCC	63,257
180	RESPIRATORY NEOPLASMS W MCC	63,015
315	OTHER CIRCULATORY SYSTEM DIAGNOSES W CC	62,140
483	MJR JNT&LMB REATTCHMNT PX UP EXT	61,562
982	EXT OR PX UNRELATED TO PRINCIPAL DX W CC	61,494
684	RENAL FAILURE W/O CC/MCC	61,388
896	ALC/DRUG ABUSE OR DEPEND W/O REHAB WMCC	61,342
863	POSTOP&POST-TRAUMATIC INFECTIONS W/OMCC	60,639
379	G.I. HEMORRHAGE W/O CC/MCC	60,636
386	INFLAMMATORY BOWEL DISEASE W CC	59,599
219	CRD VLV&OTH MJ CD/THR PX W/O CD CTH WMCC	59,326
164	MAJOR CHEST PROCEDURES W CC	58,831
299	PERIPHERAL VASCULAR DISORDERS W MCC	58,036
776	POSTPARTUM & POST ABORTION DX W/O OR PX	57,262

371	MJR GI DISORDRS & PERITONEAL INFECT WMCC	57,202
946	REHABILITATION W/O CC/MCC	57,199
282	ACUTE MI, DISCHARGED ALIVE W/O CC/MCC	57,125
694	URINARY STONES W/O ESW LITHOTRIPY W/OMCC	55,323
536	FRACTURES OF HIP & PELVIS W/O MCC	55,267
4	TRCH WMV >96HR/PDXEXCFCE/MTH&NCKW/O MJOR	54,881
617	AMP LWR LIMB ENDCRN,NTRT,&MTBL DIS W CC	54,709
433	CIRRHOSIS & ALCOHOLIC HEPATITIS W CC	54,709
882	NEUROSES EXCEPT DEPRESSIVE	54,004
560	AFTERCARE,MUSCLSKELTL SYS&CONN TISS W CC	53,948
558	TENDONITIS, MYOSITIS & BURSITIS W/O MCC	53,823
445	DISORDERS OF THE BILIARY TRACT W CC	53,183
233	CORONARY BYPASS W CARDIAC CATH W MCC	53,129
580	OTHER SKIN, SUBCUT TISS & BREAST PX W CC	51,705
388	G.I. OBSTRUCTION W MCC	49,129
163	MAJOR CHEST PROCEDURES W MCC	48,529
864	FEVER	48,430
880	ACUTE ADJSTMNT RXN&PSYCHOSOCIAL DYSFNCTN	47,564
468	REVISION HIP/KNEE REPLACEMENT W/OCC/MCC	47,462
375	DIGESTIVE MALIGNANCY W CC	47,375
242	PERMANENT CARDIAC PACEMAKER IMPLANT WMCC	46,351
473	CERVICAL SPINAL FUSION W/O CC/MCC	46,280
395	OTHER DIGESTIVE SYSTEM DX W/O CC/MCC	46,204
327	STOMACH, ESOPHAGEAL & DUODENAL PROC W CC	46,010
91	OTHER DISORDERS OF NERVOUS SYSTEM W MCC	45,794
336	PERITONEAL ADHESIOLYSIS W CC	45,555
669	TRANSURETHRAL PROCEDURES W CC	45,397
862	POSTOP & POST-TRAUMATIC INFECTIONS W MCC	45,235
71	NONSPECIFC CEREBROVASCULAR DISORDERS WCC	45,115
435	MALIG OF HEPATOBILIARY SYS/PANCREAS WMCC	44,733
56	DEGENERATIVE NERVOUS SYSTM DISORDRS WMCC	44,687

326	STOMACH, ESOPHAGEAL & DUODENAL PROC WMCC	44,169
86	TRAUMATIC STUPOR & COMA, COMA <1 HR W CC	44,144
920	COMPLICATIONS OF TREATMENT W CC	43,935
54	NERVOUS SYSTEM NEOPLASMS W MCC	43,544
249	PERC CRDVSCPX W NONDRUGELUT STENT W/OMCC	43,543
244	PERM CARDIAC PACEMAKER IMPLANT W/OCC/MCC	43,335
813	COAGULATION DISORDERS	43,312
70	NONSPEC CEREBROVASCULAR DISORDERS WMCC	43,304
462	BILAT/MULT MJR JNT PX OF LWR EXT W/OMCC	43,299
373	MJR GI DISORDRS&PERITNL INFECT W/OCC/MCC	43,162
167	OTHER RESP SYSTEM O.R. PROCEDURES W CC	42,712
181	RESPIRATORY NEOPLASMS W CC	42,163
340	APPENDECTOMY W COMPLICATED PDX W/OCC/MCC	41,955
251	PERC CRDVSC PX W/O CRNRY ART STNT W/OMCC	41,733
254	OTHER VASCULAR PROCEDURES W/O CC/MCC	40,689
644	ENDOCRINE DISORDERS W CC	40,578
149	DYSEQUILIBRIUM	40,444
581	OTH SKIN,SUBCUT TISS&BREAST PX W/OCC/MCC	40,319
857	POSTOP/POST-TRAUMA INFECT W OR PX W CC	40,241
949	AFTERCARE W CC/MCC	39,064
472	CERVICAL SPINAL FUSION W CC	38,690
27	CRANIOTMY&ENDVSC INTRCRANIALPX W/OCC/MCC	38,667
264	OTHER CIRCULATORY SYSTEM O.R. PROCEDURES	38,559
464	WND DBD&GRFT EXC HND,MSCLCNN TSS DS W CC	38,555
866	VIRAL ILLNESS W/O MCC	38,525
778	THREATENED ABORTION	38,383
235	CORONARY BYPASS W/O CARDIAC CATH W MCC	37,508
343	APPENDECTOMY W/O COMPLICTD PDX W/OCC/MCC	37,465
988	NON-EXT OR PX UNRELATED TO PRINC DX W CC	37,364
444	DISORDERS OF THE BILIARY TRACT W MCC	37,293
238	MAJOR CARDIOVASC PROCEDURES W/O MCC(V32)	37,246

908	OTHER O.R. PROCEDURES FOR INJURIES W CC	36,881
919	COMPLICATIONS OF TREATMENT W MCC	36,867
301	PERIPHERAL VASCULAR DISORDERS W/O CC/MCC	36,396
26	CRANIOTOMY&ENDOVASC INTRACRANIAL PX WCC	36,327
947	SIGNS & SYMPTOMS W MCC	36,306
554	BONE DISEASES & ARTHROPATHIES W/O MCC	36,203
556	SIGNS&SYMP MSCLSKLTL SYS&CONN TIS W/OMCC	36,160
516	OTH MUSCLOSKELT SYS&CONN TISS OR PX W CC	36,140
543	PATH FX & MSCLSKLT&CONN TISS MALIG W CC	35,993
660	KIDNEY&URETER PX FOR NON-NEOPLASM W CC	35,728
165	MAJOR CHEST PROCEDURES W/O CC/MCC	35,463
200	PNEUMOTHORAX W CC	35,435
328	STOMACH, ESOPH & DUODENAL PROC W/OCC/MCC	35,201
788	CESAREAN SECT W/O STERILIZATION W/O CC/M	35,000
895	ALCOHOL/DRUG ABUSE OR DEPENDENCE W REHAB	34,571
551	MEDICAL BACK PROBLEMS W MCC	34,327
354	HERNIA PX EXCEPT INGUINAL & FEMORAL W CC	34,039
374	DIGESTIVE MALIGNANCY W MCC	33,334
605	SKIN, SUBCUT TISS & BREAST TRAUMA W/OMCC	33,250
894	ALCOHOL/DRUG ABUSE OR DEPENDENCE,LEFTAMA	32,859
23	CRNMJRDVIMPL/ACTCPLXCNS PDXWMCC/CHMO IMP	32,828
303	ATHEROSCLEROSIS W/O MCC	32,479
87	TRAUM STUPOR&COMA, COMA <1 HR W/OCC/MCC	32,376
789	NEONATES,DIED/TRANS ANTHR ACUTE CARE FAC	32,128
673	OTHER KIDNEY & URINARY TRACT PX W MCC	32,008
387	INFLAMMATORY BOWEL DISEASE W/O CC/MCC	31,394
907	OTHER O.R. PROCEDURES FOR INJURIES W MCC	31,243
206	OTHER RESPIRATORY SYSTEM DX W/O MCC	31,037
987	NON-EXT OR PX UNRELATED TO PRINC DX WMCC	30,988
216	CRD VLV&OTH MJ CRD/THR PX W CRD CTH WMCC	30,734
561	AFTERCRE,MSCLSKLTL SYS&CON TIS W/OCC/MCC	30,667

85	TRAUMATIC STUPOR & COMA, COMA <1 HR WMCC	30,605
270	OTHER MAJ CARDIO PX W MCC	30,594
355	HERNIA PX EXC INGUINAL&FEMORAL W/OCC/MCC	30,511
93	OTH DISORDERS OF NERVOUS SYSTM W/OCC/MCC	30,036
436	MALIG OF HEPATOBILIARY SYS/PANCREAS W CC	29,977
62	ACUTE ISCHM STROKE W THROMBLYTC AGNT WCC	29,936
204	RESPIRATORY SIGNS & SYMPTOMS	29,845
446	DISORDERS OF THE BILIARY TRACT W/OCC/MCC	29,474
337	PERITONEAL ADHESIOLYSIS W/O CC/MCC	29,458
186	PLEURAL EFFUSION W MCC	29,225
643	ENDOCRINE DISORDERS W MCC	28,489
856	POSTOP/POST-TRAUMA INFECT W OR PX WMCC	28,404
357	OTHER DIGESTIVE SYSTEM O.R. PX W CC	28,199
237	MAJOR CARDIOVASC PROCEDURES W MCC(V32)	28,165
184	MAJOR CHEST TRAUMA W CC	27,836
657	KIDNEY & URETER PX FOR NEOPLASM W CC	27,566
455	COMBND ANTR/POSTR SPINAL FUSN W/OCC/MCC	27,392
571	SKIN DEBRIDEMENT W CC	27,011
454	COMBINED ANTR/POSTR SPINAL FUSION W CC	26,901
240	AMP FR CIRC SYS DSRDR EXC UP LMB&TOE WCC	26,704
501	SOFT TISSUE PROCEDURES W CC	26,500
607	MINOR SKIN DISORDERS W/O MCC	26,423
517	OTH MSCLSKLT SYS&CNN TS OR PX W/O CC/MCC	26,352
840	LYMPHOMA & NON-ACUTE LEUKEMIA W MCC	25,855
974	HIV W MAJOR RELATED CONDITION W MCC	25,748
356	OTHER DIGESTIVE SYSTEM O.R. PX W MCC	25,671
808	MJR HEM/IMM DX EXC SCKL CL CRS&COAG WMCC	25,644
658	KIDNEY&URETER PX FOR NEOPLASM W/OCC/MCC	25,621
708	MAJOR MALE PELVIC PROCEDURES W/O CC/MCC	25,507
956	LIMB REATT,HIP&FEMUR PX MULT SIG TRAUMA	25,432
239	AMP FR CIRC SYS DSRDR EXC UP LMB&TO WMCC	24,855

841	LYMPHOMA & NON-ACUTE LEUKEMIA W CC	24,768
304	HYPERTENSION W MCC	24,656
271	OTHER MAJ CARDIO PX W CC	24,537
41	PR/CRN NRV&OTH NRVSYS PXWCC/PRIPH NROSTM	24,321
339	APPENDECTOMY W COMPLICATED PDX W CC	24,305
728	INFLAMMATION MALE REPRODUCTVE SYS W/OMCC	24,229
620	O.R. PROCEDURES FOR OBESITY W CC	24,096
886	BEHAVIORAL & DEVELOPMENTAL DISORDERS	23,842
478	BX MUSCULOSKELTL SYS&CONNECTVE TISS W CC	23,485
883	DISORDERS OF PERSONALITY&IMPULSE CONTROL	23,417
179	RESPIRATRY INFECT&INFLAMMATNS W/OCC/MCC	23,139
39	EXTRACRANIAL PROCEDURES W/O CC/MCC	22,484
248	PRC CRDVSCPX W NNDRGELT ST WMCC /4+VS/ST	22,432
196	INTERSTITIAL LUNG DISEASE W MCC	22,233
492	LWR EXT&HUMER PX EXC HIP,FOOT,FEMUR WMCC	22,147
520	BCK/NCK PX EXC SPIN FUS W/OCC/MCC	22,140
227	CARD DEFIB IMPLANT W/O CARD CATH W/OMCC	21,905
661	KIDNEY&URETER PX NONNEOPLASM W/OCC/MCC	21,832
964	OTHER MULTIPLE SIGNIFICANT TRAUMA W CC	21,697
629	OTH ENDOCRINE, NUTRIT & METAB OR PX W CC	21,582
187	PLEURAL EFFUSION W CC	21,548
38	EXTRACRANIAL PROCEDURES W CC	21,530
73	CRANIAL&PERIPHERAL NERVE DISORDERS WMCC	21,324
274	PERCUTANEOUS INTRACARDIAC PX W/O MCC	21,275
381	COMPLICATED PEPTIC ULCER W CC	21,160
267	ENDOVASC CARD VALV REPL W/OMCC	20,838
519	BCK/NCK PX EXC SPIN FUS W CC	20,821
546	CONNECTIVE TISSUE DISORDERS W CC	20,705
623	SKN GRFT&WND DBRD ENDC,NTRT&MTB DIS W CC	20,641
674	OTHER KIDNEY & URINARY TRACT PX W CC	20,425
951	OTHER FACTORS INFLUENCING HEALTH STATUS	20,408

335	PERITONEAL ADHESIOLYSIS W MCC	20,211
205	OTHER RESPIRATORY SYSTEM DIAGNOSES W MCC	20,156
55	NERVOUS SYSTEM NEOPLASMS W/O MCC	20,083
384	UNCOMPLICATED PEPTIC ULCER W/O MCC	19,868
782	OTH ANTEPARTUM DX W/O MEDICAL COMPLICATN	19,726
958	OTH OR PX MULT SIGNIFICANT TRAUMA W CC	19,709
250	PERC CRDVSC PX W/O CORNRY ART STENT WMCC	19,663
957	OTH OR PX MULT SIGNIFICANT TRAUMA W MCC	19,552
283	ACUTE MYOCARDIAL INFARCTION, EXPIRD WMCC	19,031
199	PNEUMOTHORAX W MCC	18,916
443	DSDR LVR EXC MALG,CIRR,ALCHEPA W/OCC/MCC	18,850
806	VAG DELIV W/O STERILIZATION/D&C W CC	18,706
369	MAJOR ESOPHAGEAL DISORDERS W CC	18,596
645	ENDOCRINE DISORDERS W/O CC/MCC	18,374
565	OTH MUSCULOSKELETL SYS&CONN TISS DX W CC	18,288
983	EXT OR PX UNRELATE TO PRIN DX W/O CC/MCC	18,197
463	WND DBD&GRFT EXC HND,MSCLCNN TSS DS WMCC	18,146
542	PATH FX & MSCLSKLT&CONN TISS MALIG WMCC	18,112
60	MULT SCLERSIS&CEREBELLR ATAXIA W/OCC/MCC	17,864
559	AFTERCARE,MUSCLSKELTL SYS&CONN TISS WMCC	17,736
579	OTHER SKIN, SUBCUT TISS & BREAST PX WMCC	17,649
266	ENDOVASC CARD VALV REPL WMCC	17,481
696	KIDNEY&URINARY TRACT SIGNS&SYMPT W/OMCC	17,319
406	PANCREAS, LIVER & SHUNT PROCEDURES W CC	17,212
535	FRACTURES OF HIP & PELVIS W MCC	17,209
491	BACK&NECK PX EXC SPINAL FUSION W/OCC/MCC	17,182
405	PANCREAS, LIVER & SHUNT PROCEDURES W MCC	17,060
158	DENTAL & ORAL DISEASES W CC	17,012
29	SPINAL PX WCC OR SPINAL NEUROSTIMULATORS	16,938
459	SPINAL FUSION EXCEPT CERVICAL W MCC	16,677
975	HIV W MAJOR RELATED CONDITION W CC	16,637

40	PERIPH/CRANIAL NERV&OTH NERV SYS PX WMCC	16,532
201	PNEUMOTHORAX W/O CC/MCC	16,528
155	OTHER EAR, NOSE, MOUTH & THROAT DX W CC	16,262
909	OTHER O.R. PX FOR INJURIES W/O CC/MCC	16,111
700	OTHER KIDNEY&URINARY TRACT DX W/OCC/MCC	16,073
152	OTITIS MEDIA & URI W MCC	15,637
834	ACUTE LEUKEMIA W/O MAJOR O.R. PX W MCC	15,415
226	CARDIAC DEFIB IMPLANT W/O CARD CATH WMCC	15,319
562	FX,SPN,STN&DISL EX FMR,HP,PLVS&THGH WMCC	15,288
457	SP FS EXC CRV W SP CRV/MLG/INF /9+FS WCC	15,193
846	CHEMO W/O ACUTE LEUKEMIA SECONDR DX WMCC	15,050
229	OTHER CARDIOTHORACIC PROCEDURES W/O MCC	14,949
316	OTHER CIRC SYSTEM DIAGNOSES W/O CC/MCC	14,940
348	ANAL & STOMAL PROCEDURES W CC	14,877
83	TRAUMATIC STUPOR & COMA, COMA >1 HR WCC	14,870
540	OSTEOMYELITIS W CC	14,845
59	MULTIPLE SCLEROSIS&CEREBELLAR ATAXIA WCC	14,702
61	ACUTE ISCHM STROKE W THROMBLYT AGNT WMCC	14,693
652	KIDNEY TRANSPLANT	14,644
502	SOFT TISSUE PROCEDURES W/O CC/MCC	14,626
345	MINOR SMALL & LARGE BOWEL PX W CC	14,475
490	BCK&NCKPX EXC SFS WCC/MCC OR DSC DV/NRST	14,444
221	CD VLV&OT MJ CD/TRPXW/O CD CTH W/OCC/MCC	14,419
572	SKIN DEBRIDEMENT W/O CC/MCC	14,344
342	APPENDECTOMY W/O COMPLICATED PDX W CC	14,120
839	CHEMO W ACUTE LEUKEMIA AS SDX W/O CC/MCC	14,001
921	COMPLICATIONS OF TREATMENT W/O CC/MCC	13,942
787	CESAREAN SECT W/O STERILIZATION W CC	13,927
515	OTH MUSCLOSKELT SYS&CONN TISS OR PX WMCC	13,785
737	UTER&ADNX PX OVARIAN/ADNEXAL MALIG W CC	13,779
707	MAJOR MALE PELVIC PROCEDURES W CC/MCC	13,762

76	VIRAL MENINGITIS W/O CC/MCC	13,704
916	ALLERGIC REACTIONS W/O MCC	13,661
183	MAJOR CHEST TRAUMA W MCC	13,544
82	TRAUMATIC STUPOR & COMA, COMA >1 HR WMCC	13,427
475	AMP FOR MSCLSKLTL SYS&CONN TISS DIS W CC	13,306
504	FOOT PROCEDURES W CC	13,302
512	SHLDR,ELBW/FRM PX,EX MJR JNT PXW/OCC/MCC	13,271
570	SKIN DEBRIDEMENT W MCC	13,246
769	POSTPARTUM & POST ABORTION DX W OR PX	13,190
228	OTHER CARDIOTHORACIC PROCEDURES W MCC	13,170
385	INFLAMMATORY BOWEL DISEASE W MCC	13,164
545	CONNECTIVE TISSUE DISORDERS W MCC	13,008
865	VIRAL ILLNESS W MCC	12,892
217	CRD VLV&OTH MJ CRD/THR PX W CRD CTH WCC	12,884
415	CHOLECYSTCTMY EXC BY LAPSCP W/O CDE W CC	12,849
269	AORTIC&HRT ASST PX EXC PULSN BLN W/O MCC	12,755
380	COMPLICATED PEPTIC ULCER W MCC	12,744
557	TENDONITIS, MYOSITIS & BURSITIS W MCC	12,723
592	SKIN ULCERS W MCC	12,636
596	MAJOR SKIN DISORDERS W/O MCC	12,587
511	SHLDR,ELBW/FRARM PX,EXC MJR JNT PX WCC	12,569
659	KIDNEY&URETER PX FOR NON-NEOPLASM W MCC	12,392
471	CERVICAL SPINAL FUSION W MCC	12,355
488	KNEE PX W/O PDX OF INFECTION W CC/MCC	12,344
125	OTHER DISORDERS OF THE EYE W/O MCC	12,338
815	RETICULOENDOTHELIAL & IMM DISORDERS W CC	12,261
496	LCL EXC&RMVL INT FIX DV EXC HIP&FMR WCC	12,181
867	OTH INFECTIOUS & PARASITIC DIS DX W MCC	12,121
484	MJR JNT&LMB RTTCHMNT PX UP EXT W/OCC/MCC	12,046
72	NONSPEC CEREBROVASC DISORDERS W/OCC/MCC	12,033
346	MINOR SMALL & LARGE BOWEL PX W/O CC/MCC	11,996

989	NON-EXT OR PX UNRELATD PRIN DX W/OCC/MCC	11,942
273	PERCUTANEOUS INTRACARDIAC PX W MCC	11,928
759	INFECTNS,FEMALE REPRODUCTV SYS W/OCC/MCC	11,816
134	OTHER ENT & MOUTH O.R. PX W/O CC/MCC	11,767
593	SKIN ULCERS W CC	11,742
465	WD DBD&GRFT EXC HND,MSCLCNN DS W/OCC/MCC	11,723
713	TRANSURETHRAL PROSTATECTOMY W CC/MCC	11,717
311	ANGINA PECTORIS	11,694
133	OTHER ENT & MOUTH O.R. PX W CC/MCC	11,661
654	MAJOR BLADDER PROCEDURES W CC	11,628
368	MAJOR ESOPHAGEAL DISORDERS W MCC	11,491
84	TRAUM STUPOR&COMA, COMA >1 HR W/O CC/MCC	11,426
489	KNEE PX W/O PDX OF INFECTION W/O CC/MCC	11,380
466	REVISION OF HIP OR KNEE REPLACEMENT WMCC	11,241
168	OTHER RESP SYSTEM O.R. PX W/O CC/MCC	11,208
740	UTER,ADNX PX NONOVARN/ADNEXAL MALIG W CC	11,138
37	EXTRACRANIAL PROCEDURES W MCC	11,061
810	MJR HM/IM DX EX SCKL CL CRS&CG W/OCC/MCC	11,003
349	ANAL & STOMAL PROCEDURES W/O CC/MCC	10,945
963	OTHER MULTIPLE SIGNIFICANT TRAUMA W MCC	10,821
837	CHEM W AC LKM SDX/W HGH DS CHM AGNT WMCC	10,813
197	INTERSTITIAL LUNG DISEASE W CC	10,683
760	MNSTRL&OTH FEMALE REPROD SYS DIS WCC/MCC	10,608
353	HERNIA PX EXCEPT INGUINAL & FEMORAL WMCC	10,560
547	CONNECTIVE TISSUE DISORDERS W/O CC/MCC	10,539
68	NONSPCCVA&PRCRBRL OCCL W/O INFRCT W/OMCC	10,439
755	MALIGNANCY, FEMALE REPRODUCTIVE SYS W CC	10,386
30	SPINAL PROCEDURES W/O CC/MCC	10,373
668	TRANSURETHRAL PROCEDURES W MCC	10,352
24	CRN WMJRDEV IMPL/ACUTE CPLXCNS PDXW/OMCC	10,314
914	TRAUMATIC INJURY W/O MCC	10,282

628	OTH ENDOCRINE, NUTRIT & METAB OR PX WMCC	10,203
758	INFECTIONS, FEMALE REPRODUCTIVE SYS WCC	10,202
768	VAGINAL DELV W OR PX EXC STERIL &/OR D&C	10,175
123	NEUROLOGICAL EYE DISORDERS	10,167
156	OTHER ENT & MOUTH DIAGNOSES W/O CC/MCC	10,077
131	CRANIAL/FACIAL PROCEDURES W CC/MCC	9,934
477	BX MUSCULOSKELTL SYS&CONNECTVE TISS WMCC	9,837
950	AFTERCARE W/O CC/MCC	9,824
333	RECTAL RESECTION W CC	9,820
935	NON-EXTENSIVE BURNS	9,660
656	KIDNEY & URETER PX FOR NEOPLASM W MCC	9,626
351	INGUINAL & FEMORAL HERNIA PX W CC	9,613
307	CARDIAC CONGENTL & VALV DISORDERS W/OMCC	9,533
868	OTH INFECTIOUS & PARASITIC DIS DX W CC	9,511
513	HND/WRST PX, EXC MJR THMB/JNT PX WCC/MCC	9,457
642	INBORN AND OTHER DISORDERS OF METABOLISM	9,392
564	OTH MUSCULOSKELETL SYS&CONN TISS DX WMCC	9,379
616	AMP LWR LIMB ENDCRN,NTRT,&MTBL DIS WMCC	9,368
824	LYMPHOMA&NONACUTE LEUKM W OTH OR PX W CC	9,339
539	OSTEOMYELITIS W MCC	9,330
302	ATHEROSCLEROSIS W MCC	9,233
977	HIV W OR W/O OTHER RELATED CONDITION	9,227
486	KNEE PROCEDURES W PDX OF INFECTION W CC	9,201
474	AMP FOR MSCLSKLTL SYS&CONN TISS DIS WMCC	9,178
544	PATH FX & MSCLSKLT&CN TS MALIG W/OCC/MCC	9,171
256	UP LMB&TOE AMP FOR CIRC SYS DISORDER WCC	9,096
416	CHCYTCTMY EXC BY LPSCP W/O CDE W/OCC/MCC	9,095
28	SPINAL PROCEDURES W MCC	9,093
458	SPFS EXC CR W CR/MLG/INF/9+FS W/OCC/MCC	9,082
132	CRANIAL/FACIAL PROCEDURES W/O CC/MCC	9,025
352	INGUINAL & FEMORAL HERNIA PX W/O CC/MCC	8,957

75	VIRAL MENINGITIS W CC/MCC	8,955
306	CARDIAC CONGENITAL & VALV DISORDERS WMCC	8,800
224	CRD DFB IMP W CRD CTH W/OAMI/HF/SHK WMCC	8,791
358	OTHER DIGESTIVE SYSTEM O.R. PX W/OCC/MCC	8,765
414	CHOLECYSTCTMY EXC BY LAPSCP W/O CDE WMCC	8,640
159	DENTAL & ORAL DISEASES W/O CC/MCC	8,596
505	FOOT PROCEDURES W/O CC/MCC	8,589
902	WOUND DEBRIDEMENTS FOR INJURIES W CC	8,552
835	ACUTE LEUKEMIA W/O MAJOR O.R. PX W CC	8,522
20	INTRCRANIAL VASC PX WPDX HEMORRHAGE WMCC	8,520
670	TRANSURETHRAL PROCEDURES W/O CC/MCC	8,383
915	ALLERGIC REACTIONS W MCC	8,364
940	OR PX W DX OF OTH CONT W HEALTH SRV W CC	8,309
500	SOFT TISSUE PROCEDURES W MCC	8,267
261	CARD PACEMKR REV EXC DEV REPLACEMNT WCC	8,223
334	RECTAL RESECTION W/O CC/MCC	8,188
555	SIGNS&SYMP MSCLSKLTL SYS&CONN TISS WMCC	8,102
97	NONBCT INFCT NRVSYS EXC VIRL MNNGTS WMCC	8,093
42	PERIPH/CRAN NERV&OTH NERVSYS PXW/OCC/MCC	8,076
78	HYPERTENSIVE ENCEPHALOPATHY W CC	8,040
687	KIDNEY & URINARY TRACT NEOPLASMS W CC	8,013
838	CHM W ACT LKM SDX WCC OR HGH DS CHM AGNT	7,891
63	ACT ISCHM STRK W THRMBLY AGNT W/OCC/MCC	7,878
32	VENTRICULAR SHUNT PROCEDURES W CC	7,870
741	UTR,ADNXPX NONOVAR/ADNXL MALIG W/OCC/MCC	7,845
151	EPISTAXIS W/O MCC	7,816
923	OTH INJRY,POISONING&TOXC EFFCT DX W/OMCC	7,808
693	URINARY STONES W/O ESW LITHOTRIPSY W MCC	7,749
585	BRST BX, LOC EXCIS&OTH BRST PX W/OCC/MCC	7,740
95	BACTERIAL&TB INFECTIONS OF NERV SYS WCC	7,740
614	ADRENAL & PITUITARY PROCEDURES W CC/MCC	7,695

816	RETICULOENDOTHEL&IMM DISORDERS W/OCC/MCC	7,693
823	LYMPHOMA&NONACUTE LEUKM W OTH OR PX WMCC	7,666
272	OTHER MAJ CARDIO PX W/O CC/MCC	7,614
604	SKIN, SUBCUT TISS & BREAST TRAUMA WMCC	7,613
842	LYMPHOMA & NON-ACUTE LEUKEMIA W/O CC/MCC	7,529
922	OTH INJURY,POISONING&TOXC EFFECT DX WMCC	7,455
744	D&C,CONIZATN,LAP&TUBAL INTERRUPT WCC/MCC	7,351
479	BX MUSCULOSKLTL SYS&CNNCTV TSS W/OCC/MCC	7,340
407	PANCREAS, LIVER & SHUNT PX W/O CC/MCC	7,323
225	CRD DFB IP W CRD CTH W/OAMI/HF/SK W/OMCC	7,309
858	POSTOP/POSTTRAUM INFECT WOR PX W/OCC/MCC	7,294
777	ECTOPIC PREGNANCY	7,273
583	MASTECTOMY FOR MALIGNANCY W/O CC/MCC	7,201
98	NONBCT INFCT NRV SYS EXC VIRL MNNGTS WCC	7,191
844	OTH MYELOPROLF DIS/PRLY DIFF NEO DX W CC	7,069
185	MAJOR CHEST TRAUMA W/O CC/MCC	7,026
89	CONCUSSION W CC	7,017
11	TRACH FOR FACE,MOUTH & NECK DX W MCC	7,003
497	LCL EXC&RM INT FX DV EX HP&FMR W/OCC/MCC	6,832
94	BACTERIAL&TB INFECTIONS OF NERV SYS WMCC	6,807
518	BCK/NCK PX EXC SPIN FUS WMCC/DEV/NRV STI	6,775
288	ACUTE & SUBACUTE ENDOCARDITIS W MCC	6,747
601	NON-MALIGNANT BREAST DISORDERS W/OCC/MCC	6,742
534	FRACTURES OF FEMUR W/O MCC	6,724
102	HEADACHES W MCC	6,694
215	OTHER HEART ASSIST SYSTEM IMPLANT	6,674
453	COMBINED ANTR/POSTR SPINAL FUSION W MCC	6,673
154	OTHER EAR, NOSE, MOUTH & THROAT DX W MCC	6,592
16	AUTOLOGOUS BONE MARROW TRNSPLNT W CC/MCC	6,520
99	NONBCT INFCT NRV EX VRL MNNGTS W/OCC/MCC	6,490
137	MOUTH PROCEDURES W CC/MCC	6,419

785	CESAREAN SECT W STERILIZATION W/O CC/MCC	6,413
382	COMPLICATED PEPTIC ULCER W/O CC/MCC	6,388
553	BONE DISEASES & ARTHROPATHIES W MCC	6,382
843	OTH MYELOPROLF DIS/PRLY DIFF NEO DX WMCC	6,371
420	HEPATOBILIARY DIAGNOSTIC PROCEDURES WMCC	6,361
245	AICD GENERATOR PROCEDURES	6,355
566	OTH MUSCLSKLTL SYS&CONN TIS DX W/OCC/MCC	6,244
779	ABORTION W/O D&C	6,238
157	DENTAL & ORAL DISEASES W MCC	6,233
726	BENIGN PROSTATIC HYPERTROPHY W/O MCC	6,225
734	PELV EVSC,RAD HYSTRCT&RAD VLVCT WCC/MCC	5,983
260	CARD PACEMKR REV EXC DEV REPLACEMNT WMCC	5,973
746	VAGINA, CERVIX & VULVA PX W CC/MCC	5,963
770	ABORT W D&C,ASP CURETTAGE OR HYSTEROTOMY	5,960
514	HND/WRSTPX, EXC MJR THMB/JNTPX W/OCC/MCC	5,944
12	TRACH FOR FACE,MOUTH & NECK DX W CC	5,929
296	CARDIAC ARREST, UNEXPLAINED W MCC	5,921
714	TRANSURETHRAL PROSTATECTOMY W/O CC/MCC	5,901
598	MALIGNANT BREAST DISORDERS W CC	5,844
408	BIL TRCT PX EXC CHLCYST W/W/O CDE WMCC	5,837
52	SPINAL DISORDERS & INJURIES W CC/MCC	5,826
268	AORTIC&HRT ASST PX EXC PULSN BLN W MCC	5,795
761	MNSTRL&OTH FEM REPROD SYS DIS W/OCC/MCC	5,759
255	UP LMB&TOE AMP FOR CIRC SYS DISORDR WMCC	5,749
754	MALIGNANCY, FEMALE REPRODUCTIVE SYS WMCC	5,742
829	MYLPR DS/PRLY DIF NPL W OTH OR PXWCC/MCC	5,721
222	CRD DFB IMPL W CRD CTH WAMI/HF/SHCK WMCC	5,687
33	VENTRICULAR SHUNT PROCEDURES W/O CC/MCC	5,681
814	RETICULOENDOTHELIAL & IMM DISORDERS WMCC	5,567
749	OTH FEMALE REPRODUCTVE SYS OR PX WCC/MCC	5,532
549	SEPTIC ARTHRITIS W CC	5,523

786	CESAREAN SECT W/O STERILIZATION W MCC	5,517
573	SKIN GRAFT FOR ULCER/CELLULITIS W MCC	5,491
421	HEPATOBILIARY DIAGNOSTIC PROCEDURES W CC	5,489
965	OTHER MULT SIGNIFICANT TRAUMA W/O CC/MCC	5,489
627	THYROID,PARATHY&THYROGLOSS PX W/O CC/MCC	5,480
96	BCTERIAL&TB INFECT OF NERV SYS W/OCC/MCC	5,469
456	SPFS EXC CRV W SP CRV/MLG/INF /9+FS WMCC	5,469
77	HYPERTENSIVE ENCEPHALOPATHY W MCC	5,465
904	SKIN GRAFTS FOR INJURIES W CC/MCC	5,435
606	MINOR SKIN DISORDERS W MCC	5,424
805	VAG DELIV W/O STERILIZATION/D&C W MCC	5,286
376	DIGESTIVE MALIGNANCY W/O CC/MCC	5,262
600	NON-MALIGNANT BREAST DISORDERS W CC/MCC	5,251
541	OSTEOMYELITIS W/O CC/MCC	5,226
338	APPENDECTOMY W COMPLICATED PDX W MCC	5,221
582	MASTECTOMY FOR MALIGNANCY W CC/MCC	5,197
81	NONTRAUMATIC STUPOR & COMA W/O MCC	5,185
747	VAGINA, CERVIX & VULVA PX W/O CC/MCC	5,105
409	BIL TRCT PX EXC CHLCYST W/W/O CDE WCC	5,070
1	HEART TRNSPLT/IMPLT HEART AST SYS WMCC	5,060
941	OR PX W DX OTH CNT W HLTH SRV W/OCC/MCC	4,986
615	ADRENAL & PITUITARY PROCEDURES W/OCC/MCC	4,955
129	MJR HEAD&NECK PX WCC/MCC OR MAJOR DEVICE	4,939
622	SKN GRFT&WND DBRD ENDC,NTRT&MTB DIS WMCC	4,920
626	THYROID,PARATHYROID&THYROGLOSSAL PX W CC	4,886
939	OR PX W DX OF OTH CONT W HEALTH SRV WMCC	4,843
577	SKIN GRAFT EXC FOR ULCER/CELLULITIS W CC	4,843
578	SKIN GRFT EXC FR ULCR/CELLITIS WO CC/MCC	4,840
231	CORONARY BYPASS W PTCA W MCC	4,832
748	FEMALE REPRODUCTVE SYS RECONSTRUCTIVE PX	4,791
803	OTH OR PX BLOOD&BLOOD FORMNG ORGANS W CC	4,776

906	HAND PROCEDURES FOR INJURIES	4,760
686	KIDNEY & URINARY TRACT NEOPLASMS W MCC	4,756
827	MYLPRLF DS/PRLY DIFF NPL W MJ OR PX W CC	4,731
653	MAJOR BLADDER PROCEDURES W MCC	4,727
188	PLEURAL EFFUSION W/O CC/MCC	4,669
31	VENTRICULAR SHUNT PROCEDURES W MCC	4,631
727	INFLAMMATION MALE REPRODUCTIVE SYS WMCC	4,571
876	O.R. PX W PRINCIPAL DX OF MENTAL ILLNESS	4,566
597	MALIGNANT BREAST DISORDERS W MCC	4,543
976	HIV W MAJOR RELATED CONDITION W/O CC/MCC	4,497
423	OTHER HEPATOBILIARY/PANCREAS OR PX W MCC	4,481
347	ANAL & STOMAL PROCEDURES W MCC	4,447
738	UTER&ADNX PX OVARN/ADNXL MALIG W/OCC/MCC	4,355
232	CORONARY BYPASS W PTCA W/O MCC	4,288
821	LYMPHOMA & LEUKEMIA W MAJOR OR PX W CC	4,263
344	MINOR SMALL & LARGE BOWEL PX W MCC	4,217
182	RESPIRATORY NEOPLASMS W/O CC/MCC	4,205
90	CONCUSSION W/O CC/MCC	4,153
350	INGUINAL & FEMORAL HERNIA PX W MCC	4,147
723	MALIGNANCY, MALE REPRODUCTIVE SYS W CC	4,126
487	KNEE PX W PDX OF INFECTION W/O CC/MCC	4,075
437	MALIG OF HEPATOBIL SYS/PANCRS W/OCC/MCC	4,071
584	BREAST BX, LOC EXCIS&OTH BRST PX WCC/MCC	4,069
574	SKIN GRAFT FOR ULCER/CELLULITIS W CC	4,054
955	CRANIOTOMY FOR MULT SIGNIFICANT TRAUMA	4,008
655	MAJOR BLADDER PROCEDURES W/O CC/MCC	4,007
848	CHEMO W/O ACUTE LEUKEMIA SECDX W/OCC/MCC	3,967
58	MULTIPLE SCLEROSIS&CEREBELLR ATAXIA WMCC	3,957
869	OTH INFECT&PARASITIC DIS DX W/O CC/MCC	3,947
289	ACUTE & SUBACUTE ENDOCARDITIS W CC	3,944
383	UNCOMPLICATED PEPTIC ULCER W MCC	3,935

820	LYMPHOMA & LEUKEMIA W MAJOR OR PX W MCC	3,922
619	O.R. PROCEDURES FOR OBESITY W MCC	3,864
14	ALLOGENEIC BONE MARROW TRANSPLANT	3,863
341	APPENDECTOMY W/O COMPLICATED PDX W MCC	3,858
198	INTERSTITIAL LUNG DISEASE W/O CC/MCC	3,824
510	SHLDR,ELBW/FRARM PX,EXC MJR JNT PX WMCC	3,788
35	CAROTID ARTERY STENT PROCEDURE W CC	3,781
498	LCL EXC&RM INT FX DV OF HIP&FMR WCC/MCC	3,757
691	URINARY STONES W ESW LITHOTRIPSY WCC/MCC	3,693
147	EAR, NOSE, MOUTH & THROAT MALIGNANCY WCC	3,690
666	PROSTATECTOMY W CC	3,643
503	FOOT PROCEDURES W MCC	3,576
901	WOUND DEBRIDEMENTS FOR INJURIES W MCC	3,573
485	KNEE PROCEDURES W PDX OF INFECTION W MCC	3,539
695	KIDNEY&URINARY TRACT SIGNS&SYMPTOMS WMCC	3,530
757	INFECTIONS, FEMALE REPRODUCTIVE SYS WMCC	3,528
259	CARDIAC PACEMAKER DEV REPLACEMNT W/OMCC	3,525
495	LCL EXC&RMVL INT FIX DV EXC HIP&FMR WMCC	3,491
223	CRD DFB IMP W CRD CTH WAMI/HF/SHK W/OMCC	3,491
663	MINOR BLADDER PROCEDURES W CC	3,412
146	EAR, NOSE, MOUTH&THROAT MALIGNANCY WMCC	3,397
887	OTHER MENTAL DISORDER DIAGNOSES	3,380
262	CRD PCEMKR REV EXC DEV RPLCMNT W/OCC/MCC	3,375
928	FUL THCK BRN W SKN GRFT/INHL INJ WCC/MCC	3,368
434	CIRRHOSIS&ALCOHOLIC HEPATITIS W/OCC/MCC	3,365
370	MAJOR ESOPHAGEAL DISORDERS W/O CC/MCC	3,355
959	OTH OR PX MULT SIGNIF TRAUMA W/O CC/MCC	3,340
138	MOUTH PROCEDURES W/O CC/MCC	3,338
903	WOUND DEBRIDEMENTS INJURIES W/OCC/MCC	3,239
21	INTRCRANIAL VASC PX W PDX HEMORRHAGE WCC	3,211
825	LYMPHM&NONACT LEUKM W OTH OR PXW/OCC/MCC	3,185

802	OTH OR PX BLOOD&BLOOD FORMNG ORGANS WMCC	3,178
506	MAJOR THUMB OR JOINT PROCEDURES	3,135
595	MAJOR SKIN DISORDERS W MCC	3,095
476	AMP FOR MSCLSKLTL SYS&CNN DIS W/OCC/MCC	3,072
735	PELV EVSC,RAD HYSTRT&RAD VLVCT W/OCC/MCC	3,066
804	OTH OR PX BLOOD&BLOOD FRM ORGN W/OCC/MCC	3,038
836	ACUTE LEUKEMIA W/O MAJOR OR PX W/OCC/MCC	3,018
736	UTER&ADNX PX OVARIAN/ADNEXAL MALIG WMCC	3,015
717	OTH MALE RPR SYS OR PX EXC MALIG WCC/MCC	2,970
780	FALSE LABOR	2,957
5	LIVER TRNSPLNT WMCC OR INTESTNL TRNSPLNT	2,949
969	HIV W EXTENSIVE O.R. PROCEDURE W MCC	2,936
88	CONCUSSION W MCC	2,906
833	OTH ANTEPART DIAG W/O O.R. PX W/O CC/MCC	2,898
241	AM FR CRCSYS DSDR EXC UP LB&TO W/OCC/MCC	2,883
121	ACUTE MAJOR EYE INFECTIONS W CC/MCC	2,853
826	MYLPRLF DS/PRLY DIFF NPL W MJ OR PX WMCC	2,804
832	OTH ANTEPART DIAG W/O O.R. PX W CC	2,776
150	EPISTAXIS W MCC	2,763
828	MYLPR DS/PRLY DIFF NPL W MJ PX W/OCC/MCC	2,732
130	MAJOR HEAD & NECK PROCEDURES W/O CC/MCC	2,660
799	SPLENECTOMY W MCC	2,646
36	CAROTID ARTERY STENT PROCEDURE W/OCC/MCC	2,606
685	ADMIT FOR RENAL DIALYSIS	2,606
784	CESAREAN SECT W STERILIZATION W CC	2,574
800	SPLENECTOMY W CC	2,558
729	OTH MALE REPRODUCTIVE SYSTEM DX W CC/MCC	2,553
550	SEPTIC ARTHRITIS W/O CC/MCC	2,546
124	OTHER DISORDERS OF THE EYE W MCC	2,525
80	NONTRAUMATIC STUPOR & COMA W MCC	2,523
67	NONSPCCVA&PRECRBRL OCCLS W/O INFRCT WMCC	2,511

822	LYMPHOMA&LEUKEMIA W MAJR OR PX W/OCC/MCC	2,499
13	TRACH FOR FACE,MOUTH&NECK DX W/O CC/MCC	2,467
711	TESTES PROCEDURES W CC/MCC	2,463
625	THYROID,PARATHYROID&THYROGLOSSL PX W MCC	2,434
745	D&C,CONIZATN,LAP&TUBAL INTRUPT W/OCC/MCC	2,404
230	OTHR CARDIOTHORACIC PROCEDURES W/OCC/MCC	2,402
739	UTER,ADNX PX NONOVARN/ADNEXAL MALIG WMCC	2,374
913	TRAUMATIC INJURY W MCC	2,344
855	INFECT &PARASITIC DIS W OR PX W/O CC/MCC	2,343
332	RECTAL RESECTION W MCC	2,324
34	CAROTID ARTERY STENT PROCEDURE W MCC	2,310
934	FULL THICK BURN W/O SKN GRFT OR INHL INJ	2,262
113	ORBITAL PROCEDURES W CC/MCC	2,246
122	ACUTE MAJOR EYE INFECTIONS W/O CC/MCC	2,203
424	OTHER HEPATOBILIARY/PANCREAS OR PX W CC	2,199
115	EXTRAOCULAR PROCEDURES EXCEPT ORBIT	2,188
722	MALIGNANCY, MALE REPRODUCTIVE SYS W MCC	2,180
79	HYPERTENSIVE ENCEPHALOPATHY W/O CC/MCC	2,134
265	AICD LEAD PROCEDURES	2,117
929	FUL THCK BRN SKN GRFT/INHL INJ W/OCC/MCC	1,982
507	MAJOR SHOULDER OR ELBOW JOINT PX WCC/MCC	1,977
537	SPRN,STRN&DSLCTN HIP,PELVS&THIGH WCC/MCC	1,968
410	BL TRT PX EXC CHLCYT W/W/O CDE W/OCC/MCC	1,963
218	CD VLV&OTH MJ CD/TH PX WCD CTH W/OCC/MCC	1,942
548	SEPTIC ARTHRITIS W MCC	1,942
533	FRACTURES OF FEMUR W MCC	1,929
801	SPLENECTOMY W/O CC/MCC	1,927
750	OTH FEMALE REPRODCTV SYS OR PX W/OCC/MCC	1,917
675	OTHER KIDNEY&URINARY TRACT PX W/O CC/MCC	1,862
412	CHOLECYSTECTOMY W C.D.E. W CC	1,819
662	MINOR BLADDER PROCEDURES W MCC	1,815

849	RADIOTHERAPY	1,789
53	SPINAL DISORDERS & INJURIES W/O CC/MCC	1,768
284	ACUTE MYOCARDIAL INFARCTION, EXPIRED WCC	1,721
999	UNGROUPABLE	1,710
709	PENIS PROCEDURES W CC/MCC	1,668
845	OTH MYLPRLF DS/PRLY DFF NEO DX W/OCC/MCC	1,641
725	BENIGN PROSTATIC HYPERTROPHY W MCC	1,634
905	SKIN GRAFTS FOR INJURIES W/O CC/MCC	1,620
258	CARDIAC PACEMAKER DEVICE REPLACEMNT WMCC	1,546
624	SKNGFT&WNDDBD EDC,NTRT&MTB DIS W/OCC/MCC	1,536
665	PROSTATECTOMY W MCC	1,512
413	CHOLECYSTECTOMY W C.D.E. W/O CC/MCC	1,502
664	MINOR BLADDER PROCEDURES W/O CC/MCC	1,479
411	CHOLECYSTECTOMY W C.D.E. W MCC	1,460
135	SINUS & MASTOID PROCEDURES W CC/MCC	1,438
461	BILAT/MULT MJR JOINT PX OF LWR EXT WMCC	1,378
575	SKIN GRAFT FR ULCER/CELLULITIS WO CC/MCC	1,374
630	OTH ENDOCRN,NUTRI&METAB OR PX W/O CC/MCC	1,372
798	VAGINAL DELIVERY W STERILIZATN/D&C W/O C	1,371
263	VEIN LIGATION & STRIPPING	1,366
688	KIDNEY&URINARY TRACT NEOPLASMS W/OCC/MCC	1,346
715	OTH MALE RPR SYS OR PX FOR MALIG WCC/MCC	1,336
671	URETHRAL PROCEDURES W CC/MCC	1,328
594	SKIN ULCERS W/O CC/MCC	1,327
667	PROSTATECTOMY W/O CC/MCC	1,288
576	SKIN GRAFT EXC FR ULCER/CELLULITIS W MCC	1,261
710	PENIS PROCEDURES W/O CC/MCC	1,238
6	LIVER TRANSPLANT W/O MCC	1,219
697	URETHRAL STRICTURE	1,206
712	TESTES PROCEDURES W/O CC/MCC	1,192
756	MALIG, FEMALE REPRODUCTIVE SYS W/OCC/MCC	1,186

294	DEEP VEIN THROMBOPHLEBITIS W CC/MCC	1,167
116	INTRAOCULAR PROCEDURES W CC/MCC	1,160
985	PROSTATIC OR PX UNRELATED PRINCP DX W CC	1,159
148	ENT & MOUTH MALIGNANCY W/O CC/MCC	1,146
830	MYLPR DS/PRLY DIF NPL W OTH PX W/OCC/MCC	1,131
499	LCL EXC&RM INT FX DV OF HP&FMR W/OCC/MCC	1,120
22	INTRCRAN VASC PX WPDX HEMORRHG W/OCC/MCC	1,102
422	HEPATOBILIARY DIAGNOSTIC PX W/O CC/MCC	1,094
831	OTH ANTEPART DIAG W/O O.R. PX W MCC	1,075
508	MAJOR SHOULDER OR ELBOW JNT PX W/OCC/MCC	1,024
114	ORBITAL PROCEDURES W/O CC/MCC	1,015
538	SPRN,STRN&DSLCTN HIP,PLVS&THGH W/OCC/MCC	993
718	OTH MALE RPR SY OR PX EXC MALG W/OCC/MCC	918
927	EXT/FULL THCK BRNS W MV>96HRS W SKN GRFT	891
984	PROSTATIC OR PX UNRELATED P DX WMCC(V34)	824
783	CESAREAN SECT W STERILIZATION W MCC	821
17	AUTOLOGOUS BONE MAROW TRNSPLNT WO CC/MCC	768
139	SALIVARY GLAND PROCEDURES	752
290	ACUTE & SUBACUTE ENDOCARDITIS W/O CC/MCC	740
730	OTH MALE REPRODUCTVE SYSTEM DX W/OCC/MCC	740
599	MALIGNANT BREAST DISORDERS W/O CC/MCC	716
257	UP LMB&TO AMP FR CRC SYS DSRDR W/OCC/MCC	639
618	AMP LWRLMB ENDCR,NTRT,&MTB DIS W/OCC/MCC	628
2	HEART TRNSPLT/IMPLT HEART AST SYS W/OMCC	591
998	PRINCIPAL DX INVALID AS DISCHARGE DX	549
692	URINARY STONES W ESW LITHOTRPY W/OCC/MCC	541
117	INTRAOCULAR PROCEDURES W/O CC/MCC	519
672	URETHRAL PROCEDURES W/O CC/MCC	519
136	SINUS & MASTOID PROCEDURES W/O CC/MCC	457
8	SIMULTANEOUS PANCREAS/KIDNEY TRANSPLANT	456
724	MALIGNANCY, MALE REPRODCTV SYS W/OCC/MCC	450

797	VAGINAL DELIVERY W STERILIZATN/D&C W CC	441
509	ARTHROSCOPY	429
425	OTH HEPBIL/PANCREAS O.R. PX W/O CC/MCC	391
7	LUNG TRANSPLANT	379
285	ACUTE MI, EXPIRED W/O CC/MCC	366
986	PROSTATIC OR PX UNRELAT PRN DX W/OCC/MCC	340
297	CARDIAC ARREST, UNEXPLAINED W CC	332
970	HIV W EXTENSIVE O.R. PROCEDURE W/O MCC	332
818	OTH ANTEPART DIAG W O.R. PX W CC	311
716	OTH MALE RPR SYS OR PX MALIG W/OCC/MCC	280
817	OTH ANTEPART DIAG W O.R. PX W MCC	238
933	EXT/FL THCK BRNS W MV>96HRS W/O SKN GRFT	234
819	OTH ANTEPART DIAG W O.R. PX W/O CC/MCC	216
295	DEEP VEIN THROMBOPHLEBITIS W/O CC/MCC	195
796	VAGINAL DELIVERY W STERILIZATN/D&C W MCC	132
298	CARDIAC ARREST, UNEXPLAINED W/O CC/MCC	106
10	PANCREAS TRANSPLANT	79

Supplementary Table 7: GRU model hyperparameters

Hyperparameter	Value
Sequence Length	100
Embedding Shape	8 dimensions
GRU hidden size	128/64/32
GRU hidden dropout	0.1/0.1/0.1
Dense hidden size	32/16
Dense Hidden dropout	0.1
Early Stopping Patience	100
