# Supplementary material for: Machine learning for patient risk stratification: standing on, or looking over, the shoulders of clinicians?
Source: NPJ Digit Med. 2021 Mar 30;4:62. doi: 10.1038/s41746-021-00426-3 (PMC8010071; doi:10.1038/s41746-021-00426-3)
Supplement: Supplementary file 2 — Reporting Summary [file 41746_2021_426_MOESM2_ESM.pdf]

nature research

Corresponding author(s): Isaac Kohane

Last updated by author(s): Jul 10, 2020

Reporting Summary

Nature Research wishes to improve the reproducibility of the work that we publish. This form provides structure for consistency and transparency in reporting. For further information on Nature Research policies, see our Editorial Policies and the Editorial Policy Checklist.

Statistics

For all statistical analyses, confirm that the following items are present in the figure legend, table legend, main text, or Methods section.

n/a	Confirmed	
☐	☒	The exact sample size (n) for each experimental group/condition, given as a discrete number and unit of measurement
☒	☐	A statement on whether measurements were taken from distinct samples or whether the same sample was measured repeatedly
☐	☒	The statistical test(s) used AND whether they are one- or two-sided<br>*Only common tests should be described solely by name; describe more complex techniques in the Methods section.*
☐	☒	A description of all covariates tested
☐	☒	A description of any assumptions or corrections, such as tests of normality and adjustment for multiple comparisons
☐	☒	A full description of the statistical parameters including central tendency (e.g. means) or other basic estimates (e.g. regression coefficient) AND variation (e.g. standard deviation) or associated estimates of uncertainty (e.g. confidence intervals)
☒	☐	For null hypothesis testing, the test statistic (e.g. F, t, r) with confidence intervals, effect sizes, degrees of freedom and P value noted<br>*Give P values as exact values whenever suitable.*
☒	☐	For Bayesian analysis, information on the choice of priors and Markov chain Monte Carlo settings
☒	☐	For hierarchical and complex designs, identification of the appropriate level for tests and full reporting of outcomes
☒	☐	Estimates of effect sizes (e.g. Cohen's d, Pearson's r), indicating how they were calculated

Our web collection on statistics for biologists contains articles on many of the points above.

Software and code

Policy information about availability of computer code

Data collection	https://products.premierinc.com/downloads/PremierHealthcareDatabaseWhitepaper.pdf
Data analysis	https://github.com/brettbj/inpatient-stratification-charges & https://figshare.com/articles/software/Source_Code_for_-_Machine_Learning_for_Patient_Risk_Stratification_Standing_on_or_looking_over_the_shoulders_of_clinicians_/12628595

For manuscripts utilizing custom algorithms or software that are central to the research but not yet described in published literature, software must be made available to editors and reviewers. We strongly encourage code deposition in a community repository (e.g. GitHub). See the Nature Research guidelines for submitting code & software for further information.

Data

Policy information about availability of data

All manuscripts must include a data availability statement. This statement should provide the following information, where applicable:

- Accession codes, unique identifiers, or web links for publicly available datasets
- A list of figures that have associated raw data
- A description of any restrictions on data availability

All data used in this study are from the Premier Healthcare Database. The Premier Healthcare Database is commercially available (https://products.premierinc.com/downloads/PremierHealthcareDatabaseWhitepaper.pdf)

Field-specific reporting

Please select the one below that is the best fit for your research. If you are not sure, read the appropriate sections before making your selection.

☒ Life sciences ☐ Behavioural & social sciences ☐ Ecological, evolutionary & environmental sciences

For a reference copy of the document with all sections, see nature.com/documents/nr-reporting-summary-flat.pdf

Life sciences study design

All studies must disclose on these points even when the disclosure is negative.

Sample size	42,896,026 multi-day inpatient admissions
Data exclusions	N/A
Replication	This was a mult-site study including data from 973 hospitals as part of the Premier Healthcare Database.
Randomization	Observational analyses with holdout validation set.
Blinding	N/A

Reporting for specific materials, systems and methods

We require information from authors about some types of materials, experimental systems and methods used in many studies. Here, indicate whether each material, system or method listed is relevant to your study. If you are not sure if a list item applies to your research, read the appropriate section before selecting a response.

Materials & experimental systems

n/a	Involved in the study	
☒	☐	Antibodies
☒	☐	Eukaryotic cell lines
☒	☐	Palaeontology and archaeology
☒	☐	Animals and other organisms
☐	☒	Human research participants
☐	☒	Clinical data
☒	☐	Dual use research of concern

Methods

n/a	Involved in the study	
☒	☐	ChIP-seq
☒	☐	Flow cytometry
☒	☐	MRI-based neuroimaging

Human research participants

Policy information about studies involving human research participants

Population characteristics	Available as Table 2.
Recruitment	All multi-day inpatient admissions from the PHD were included.
Ethics oversight	The data have been certified as de-identified via expert determination in compliance with HIPAA §164.514(b). The research was deemed to be “non-human” in consultation with the Harvard Medical School IRB. This is consistent with >600 publications using the PHD.

Note that full information on the approval of the study protocol must also be provided in the manuscript.

Clinical data

Policy information about clinical studies

All manuscripts should comply with the ICMJE guidelines for publication of clinical research and a completed CONSORT checklist must be included with all submissions.

Clinical trial registration	NA
Study protocol	Study Methods Section.
Data collection	https://products.premierinc.com/downloads/PremierHealthcareDatabaseWhitepaper.pdf<br><br>Via PHD Whitepaper -

Premier's hospital quality improvement technology solution, Quality Advisor™, contains 45 percent of all United States discharges. Premier Quality Advisor™ measures and analyzes performance to improve patient outcomes and reduce costs by integration of quality, safety and financial data. This is accomplished through benchmarking clinical and financial outcomes against peer hospitals; comparing internal and external performances in shaping best decisions; identifying care practice variations; reducing mortality, complications, readmissions and hospital-associated conditions; monitoring ongoing efforts to improve quality, resource utilization, and efficiency; and complying with regulatory reporting requirements.

The PHD is a dynamic database that is updated weekly, with data accruing since January 2000. Since 2012, there have been more than 700 hospitals contributing data each year. To date, the PHD now maintains cumulative information from more than 1,041 hospitals. The number of years that hospitals/healthcare systems have been providing data to Premier, Inc. is represented in the longitudinal graph below.

The PHD contains information on hospital and visit characteristics; admitting and attending physician specialties; healthcare payers; and patient data from standard hospital discharge billing files. This data includes demographics and disease states; admission and discharge diagnoses; information on billed services including costs at the departmental level such as medications and devices, laboratory tests performed, diagnostic and therapeutic services; microbiology test results (for a subset of hospitals); and patient disposition and discharge health status. For most data elements, less than one percent of patient records having missing information and for key elements, such as demographics and diagnostic information, less than 0.01 percent have missing data10.

Outcomes

In hospital mortality, 30-day readmission and prolonged length of stay
